# Enhancing the Detection of Long-Chain Aldehydes by Peptide-Based Biosensors Through Counter-Ion Exchange

**DOI:** 10.3390/bios16030162

**Published:** 2026-03-13

**Authors:** Tomasz Wasilewski, Damian Neubauer, Elisabete Fernandes, Rafał Kiejzik, Bartosz Szulczyński, Jacek Gębicki, Wojciech Kamysz, Marek Wojciechowski

**Affiliations:** 1 Department of Inorganic Chemistry, Faculty of Pharmacy, Medical University of Gdansk, Poland, Hallera 107, 80-416 Gdansk, Poland; 2 International Iberian Nanotechnology Laboratory, 4715-330 Braga, Portugal; 3 Department of Process Engineering and Chemical Technology, Faculty of Chemistry, Gdansk University of Technology, 11/12 G. Narutowicza Street, 80-233 Gdansk, Poland; 4 Department of Pharmaceutical Technology and Biochemistry, Chemical Faculty, Gdańsk University of Technology, Narutowicza 11/12, 80-233 Gdańsk, Poland

**Keywords:** odorant-binding proteins, Aedes aegypti, peptides, VOCs, biosensors, electronic nose, bioelectronic nose, disease diagnosis, biomarkers, lung cancer, counter-ions

## Abstract

Long-chain aldehydes, particularly nonanal, are recognized as potential volatile biomarkers of lung cancer in exhaled breath. This study investigates the influence of peptide counter-ions on the performance of QCM-based biosensors using two odorant-binding protein-derived peptides (OBPP4 and OBPP4 GSGSGS) for the selective gas-phase detection of these aldehydes. Exchanging the counter-ion from trifluoroacetate to chloride improves biosensor sensitivity and lowers the limit of detection within the set of biosensors investigated in this study. The OBPP4 GSGSGS with chloride exhibited the highest sensitivity to nonanal (0.153 Hz/ppm) and the lowest LOD (9.8 ppm), with excellent selectivity over other groups of volatiles. The novelty of this work lies in demonstrating, for the first time, that simple counter-ion exchange in synthetic peptides can significantly enhance the gas-phase binding of volatile aldehydes, classified as lung cancer biomarkers, without altering the peptide sequence, offering a straightforward and effective optimization strategy for peptide-based piezoelectric biosensors.

## 1. Introduction

Long-chain aldehydes have been classified as potential biomarkers of lung cancer (LC), reflecting oxidative stress, lipid peroxidation, and tumor-specific metabolism [1,2]. Among them, nonanal is among the most frequently reported VOCs that are elevated in LC patients’ breath, with diagnostic potential comparable to imaging or serum markers in some cohorts [3,4,5]. LC remains one of the most demanding global health issues due to its widespread prevalence and severe consequences for human health, placing a considerable burden on healthcare systems worldwide [6,7,8]. It not only has a relatively high morbidity but also stands as the leading cause of cancer-related deaths globally due to its high mortality rate. According to data from the World Health Organization (WHO), approximately 2.48 million new LC cases and 1.8 million related deaths occurred in 2022 [9]. The experience of high-income countries has shown that, unless smoking trends are reversed, developing nations are likely to face a continuous rise in LC incidence and mortality [10]. It was estimated that if morbidity and mortality remain the same as in 2022, the incidence of LC will grow to 4.62 million new cases and 3.55 million deaths by 2050 [11]. Additionally, rising levels of air pollution, driven by rapid industrialization and urban growth, further worsen respiratory and systemic health, contributing to LC mortality [12]. These alarming statistics underscore the urgency of addressing LC through risk factor analysis, timely detection, and the development of effective therapeutic approaches [13]. Currently, a range of diagnostic methods are used to identify LC, including imaging, biopsy, molecular testing, blood-based analyses, and breath diagnostics [7,14]. The high cost and complexity of these methods often limit their widespread use and suitability for frequent monitoring. This reinforces the need for non-invasive diagnostic alternatives. Additionally, the COVID-19 pandemic has further complicated lung cancer (LC) diagnosis, as its symptoms often overlap with those of respiratory infections [15]. Exhaled breath (EB) contains numerous VOCs that can reflect pathological metabolic processes, making breath analysis a promising non-invasive diagnostic approach. Therefore, there is an urgent need in the field of disease detection for the development of affordable, non-invasive, and user-friendly applications for the detection of various biomarkers in EB [5,16,17]. The development of sensitive and selective biosensors for early LC detection is a crucial step toward the development of non-invasive diagnostic tools. Conventional diagnostic methods are often time-consuming, costly, and require specialized laboratory settings, whereas biosensors offer a promising alternative due to their rapid response, high sensitivity, miniaturization potential, and suitability for point-of-care (PoC) applications.

**Figure 1:**
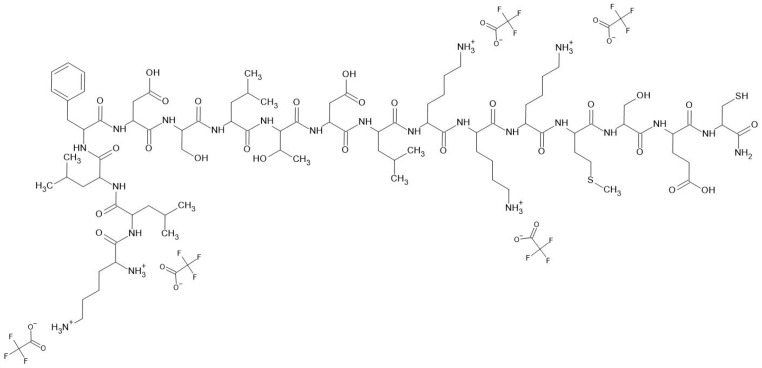
Exemplary structure of TFA^−^ and OBPP4.

A key challenge in biosensor development is optimizing peptide-analyte interactions to enhance selectivity and sensitivity. This study focuses on designing peptide-based gas biosensors derived from odorant-binding proteins (OBPs) to detect long-chain aldehydes, which are potential biomarkers of LC in exhaled breath. Two peptides of proven sensitivity to long-chain aldehydes were taken under investigation—OBPP4 (KLLFDSLTDLKKKMSEC-NH_2_) and OBPP4 with linker (KLLFDSLTDLKKKMSEGSGSGSC-NH_2_) (Figure 1). The peptide fragment, which serves as a recognition element for long-chain aldehydes, is derived from HarmOBP7, isolated from the *Helicoverpa armigera* moth. In this protein, aldehydes bind via Schiff base through the sidechains of Lys in position 123 (Lys123). The binding pocket is additionally stabilized by electrostatic interactions between Lys124 and Asp 121. Analogously, Lys125 could interact with Glu128, allowing Lys123 to interact freely with the aldehyde ligands. According to the affinity of this protein for hydrophobic linear compounds, the binding pocket also includes Leu119, Phe116, and Leu115. A more detailed analysis of the binding properties of this protein and mimicking peptides is presented in our previous studies [18,19,20]. The key amino acid identified for binding is Lys [21]. At the same time, the amino group of a Lys can be easily protonated and form a salt with a particular anion. Typically, after solid-phase peptide synthesis (SPPS), most of the peptides are trifluoroacetates due to the trifluoroacetic acid used in peptide cleavage and chromatographic purification. It has been proven that counter-ions play a critical role in modulating peptide physicochemical characteristics, conformation, solubility, and biological activity [22]. However, their performance as biorecognition elements in gas-phase biosensors has not yet been investigated. Peptides, after deposition on the gold surface of quartz crystal microbalances (QCMs) used in a biosensor, are expected to interact with aldehydes in the gas phase, but anions shielding the amino groups of Lys presumably affect the detection process. Therefore, we hypothesize that the counter-ions in peptide-based biosensors can affect ligand binding and biosensor sensitivity and that this impact differs between counter-ions. This project investigates how peptide counter-ions influence the binding affinity and response of OBP-derived peptides toward volatile biomarkers, including long-chain aldehydes. By systematically varying counter-ions, we aim to improve biosensor performance, bridging the gap between laboratory research and clinical implementation.

Both peptides were synthesized in three different salt forms, i.e., trifluoroacetic acid (TFA^−^), acetate (AcO^−^), or chloride (Cl^−^) as counter-ions. These counter-ions were selected to assess how variations in anion size, charge distribution, and hydrophobicity influence peptide–ligand interactions. For computational modeling, we assumed a 1:1 stoichiometry between the anions and the protonated amine groups (NH_3_^+^) of the peptides.

By systematically evaluating the effect of counter-ions on peptide binding, this work aims to optimize biosensor sensitivity and selectivity, ultimately facilitating the translation of laboratory-based research into practical clinical applications. To assess the effect of counter-ions, a bioelectronic nose (B-EN) was developed through a multi-step approach combining molecular docking and experimental validation. Using databases and algorithms, VOCs specific to LC were specified, and peptide sequences mimicking OBP binding sites were selected in previous studies [19,20]. These peptides were immobilized onto piezoelectric transducers to create a biosensor array. The resulting biosensors were then tested against standard mixtures of long-chain aldehydes to evaluate their basic metrological parameters, including selectivity and sensitivity (Table 2).

## 2. Materials and Method

### 2.1. Design of Peptides and Their Counter-Ions

Models of both peptide-based sensors were constructed following the stepwise procedure described in our earlier papers [19,20]. Both receptor variants were investigated: the original OBPP4 peptide as well as its modified form containing a GSGSGS linker. For in silico analyses, monolayers of peptides were deposited on a model gold surface described by the GolP-CHARMM force field [23,24,25]. Each simulation system was neutralized with suitable counter-ions: chloride (Cl^−^) or acetate (AcO^−^) or left without counter-ions in order to assess their influence on the structural organization of the peptide layer. TFA^−^ can remain associated with protonated Lys and partially “occupy” or screen the interaction region around ε-NH_3_^+^, reducing the availability of Lys to polarize/H-bond with the aldehyde carbonyl. Cl^−^ is smaller and more weakly structure-perturbing in many peptides, so it can reduce steric blocking and change local field strength at the binding energies. Changing counter-ions changes interpeptide electrostatic crosslinks and overall compactness/roughness; this alters the number, depth, and accessibility of inter-chain adsorption grooves where long-chain aldehydes preferentially reside [22,26]. In QCM-based sensing, the measured responses reflect adsorption in the whole recognition layer, not only a single residue-level interaction; thus, counter-ion-driven changes in layer packing and accessibility can shift effective binding free energy and uptake, even when docking scores differ only modestly.

**Figure 2:**
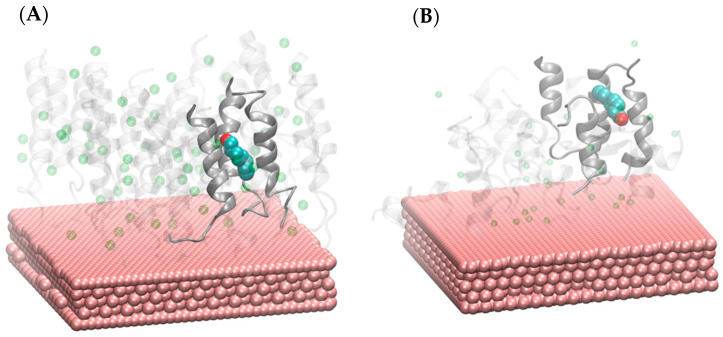
(**A**) Models of OBPP4 GSGSGS and (**B**) OBPP4 on sensors. Peptides are shown as silhouettes of their cartoon representations, with peptides directly surrounding the ligand-binding site highlighted as opaque silver ribbons. The bound ligand and chloride anions are depicted as spheres.

Resulting sensor assemblies were then subjected to 100 ns molecular dynamics simulations carried out with GROMACS 2020.4 [24,27]. The simulation trajectories were analyzed by means of clustering, and representative structures of the most abundant clusters were extracted as receptor models for docking experiments. Subsequent docking simulations were performed with AutoDock 4.2 using a blind docking approach [28]. The docking grid was defined to encompass the entire surface area of the peptide monolayer above the gold plate, thus allowing unbiased localization of potential binding sites. For each peptide–ligand pair, 50 independent docking runs were carried out, and the lowest-energy binding poses were selected for subsequent analysis. Additionally, to assess the influence of counter-ions on the compactness of peptide monolayers, the packing density index was calculated for each system. This parameter was defined as the ratio of the total van der Waals volume to the molecular volume and was determined using the Voronoia utility [29]. Figure 2 illustrates the representative MD snapshots of OBPP4 GSGSGS (A) and OBPP4 (B) monolayers on gold. It should be noted that GROMACS simulations were used to generate equilibrated sensor models (i.e., peptide monolayers on gold surfaces) and to quantify the counter-ion dependent layer organization (packing density). The reported ligand binding affinities are AutoDock scoring function estimates that are obtained by docking VOCs to their representative MD-derived cluster structures.

#### 2.1.1. Peptides Synthesis and Counter-Ion Exchange

Peptides were synthesized using a Liberty Blue™ Automated Microwave Peptide Synthesizer (CEM Corporation, Matthews, NC, USA), equipped with a fiber optic temperature probe and gas cooling system. The peptide sequence was assembled via repeated deprotection and coupling steps. The deprotection was performed using 20% piperidine in DMF in two stages: initially at 75 °C (180 W) for 15 s, followed by a second stage at 90 °C (45 W) for 55 s. After each deprotection cycle, the resin was rinsed four times with DMF. Amino acid coupling was carried out using a fivefold excess of Fmoc-protected amino acids (Fmoc-AA-OH), DIC, and Oxyma Pure, relative to the resin. Each residue was coupled twice in two microwave-assisted stages: first at 75 °C (155 W) for 15 s, and then at 90 °C (30 W) for 110 s. Upon completion of the synthesis, the resin was dried under a vacuum, and peptides were cleaved from the resin using a TFA-based cleavage cocktail (TFA:EDT:TIS: water in a 92.5:2.5:2.5:2.5 ratio, *v*/*v*/*v*/*v*) under gentle agitation for 90 min. The crude peptides were precipitated with cold diethyl ether and centrifuged. The precipitate was then dissolved in deionized water and subsequently lyophilized. Purification was achieved by reversed-phase high-performance liquid chromatography (RP-HPLC) using a Phenomenex Luna C18(2) column (10 μm, 100 Å, 100 × 21.2 mm), with a water/acetonitrile gradient and LP-chrom^®^ software (version 1.77.0.8089). The purity of synthesized peptides, confirmed to exceed 95%, was assessed by RP-HPLC (Varian, Mulgrave, VIC, Australia), while molecular identity was verified using LC-MS (Waters Alliance e2695 with Acquity QDa ESI-MS detector, Milford, MA, USA). Peptides were stored at 5 °C prior to their use in the biosensor applications.

Peptides were acquired as TFA salts. The exchange of counter-ions to Cl^−^ was carried out using a conventional method: the peptide was dissolved in a 0.1 M HCl-solution, which was allowed to incubate for 5 min and then freeze-dried. The peptides underwent further purification, this time utilizing a mobile phase (water/acetonitrile) with 0.05% HCl. This process ensured a high level of peptide purity. Peptide acetates (AcO^−^) were obtained using VariPure columns (VariPure IPE, Agilent, Santa Clara, CA, USA). The peptide TFA salt was dissolved in deionized water and applied to the prepared ion-exchange column. The VariPure column contains a resin with quaternary amine groups and a bicarbonate counter-ion; therefore, the eluted peptide is in a zwitterionic form. Peptides used in this study were found to be unstable during the VariPure resin procedure, presumably due to the oxidation of cysteine and methionine residues. Consequently, an additional chromatographic purification step was performed using acetic acid as a mobile-phase additive (0.1%, *v*/*v*). Unfortunately, despite this, the final peptide acetates had a lower purity than the TFA^−^ and Cl^−^ salts. All reagents and the volatile compounds (analytical grade) were purchased from Sigma-Aldrich (Sigma Aldrich Co., St. Louis, MO, USA).

#### 2.1.2. Immobilization

The immobilization step refers to the stable attachment of peptide molecules onto the gold surface of the quartz crystal. Achieving a well-oriented peptide layer necessitates a controlled deposition and the formation of specific chemical bonds to enhance binding interactions with volatile compounds [30]. A common and effective method involves the use of self-assembled monolayers (SAMs) on gold substrates. This is accomplished through chemisorption driven by the thiol (-SH) group of the cysteine residue that is located at the peptide’s C-terminus, which enables direct anchoring to the gold surface. Gold is particularly well-suited for this purpose due to its strong affinity for sulphur atoms, which allows for covalent bond formation without the formation of disruptive interfacial layers such as substitutional sulphide. The assembly of SAMs occurs spontaneously when thiol-functionalized peptides are introduced into the solution and allowed to interact with the gold-coated sensor surface. Thanks to its excellent biocompatibility and reliable thiol–gold interactions, gold remains the material of choice for immobilizing peptides in biosensor applications. This approach simplifies surface functionalization and supports the consistent evaluation of peptide–analyte interactions, particularly for VOCs detection [31]. Before immobilization, QCM wafers were cleaned with oxygen plasma using a previously developed method [32]. In addition, measurements of the contact angle (DSA100B Drop Shape Analyzer, KRUSS, Germany) of the gold surface of the QCM electrode were performed (Figure S2). When biomolecules adsorb onto a surface, increasing its thickness, the device detects a corresponding change in frequency [33]. As reported in the literature, the extent of deposition can be quantified by measuring the change in resonant frequency before and after peptide immobilization.

Following the Sauerbrey Equation (3), it is possible to determine an exact change in the peptide mass that is bound to the sensor [30]:

(1) $$∆F=\frac{-2F_{o}^{2}∆M}{\left[{A\left(\mu_{q}\rho_{q}\right)}^{\frac{1}{2}}\right]}$$

where $\rho_{q}\;$ and $\mu_{q}$ are the density (2.648 g·cm^−3^) and shear modulus of the quartz (2.947 × 10^11^ g·cm^−1^·s^2^), respectively, $F_{0}$ is the crystal fundamental frequency of the piezoelectric quartz crystal, A is the crystal piezoelectrically active geometrical area, which is defined by the area of the metallic film deposited on the crystal, and ΔM and ΔF are the mass and frequency changes [30,34].

Peptides were immobilized onto the gold electrodes of QCMs via the drop-casting method, a straightforward and reproducible technique that enables the application of small amounts of coating solution to create thin and uniform films [35]. The final film thickness depends on several parameters, including solution volume, peptide dispersion, concentration, solvent characteristics, and the wetting behavior (contact angle) of the solution on the substrate. Following deposition, the modified sensors were stored in a desiccator for 24 h to facilitate drying and peptide film stabilization. Peptide attachment was achieved through self-assembled monolayer (SAM) formation, leveraging the strong affinity between thiol groups (-SH) and gold surfaces. Compounds bearing thiol functionalities spontaneously organized into ordered monolayers on noble metals like gold, as they were driven by a reduction in surface energy and the formation of stable S–Au bonds [36]. The peptide solution (0.5 mg·mL^−1^ in deionized water) was deposited onto the gold electrode in 20 µL aliquots using an Eppendorf Xplorer electronic pipette (Eppendorf, Hamburg, Germany). After deposition, peptides bearing thiol groups spontaneously formed a SAM on the gold surface through S–Au interactions. All deposition procedures were conducted at room temperature following an established protocol [19,35], ensuring consistent formation of peptide coatings on the piezoelectric transducers (Figure 2).

### 2.2. VOCs Preparation and Their Analysis with Bioelectronic Nose

To identify potential breath biomarkers for LC, we conducted a review of original studies included in four relevant reviews, taking into account the diverse range of VOCs present in exhaled breath [37,38,39,40]. We selected lung cancer-related VOCs that had been reported in at least two original studies as potential breath biomarkers. VOCs at different concentration levels were prepared in Tedlar^®^ bags using a gas mixture generator as previously described [18]. Correctness of their preparation was verified with a gas chromatograph (430-GC, Bruker^®^, Bremen, Germany) according to the method that was elaborated earlier [18]. The compounds presented in Table 1 were prepared in Tedlar bags by injecting 10 µL of each VOC, which was then followed by desorption.

Based on previous research, the selected peptide sequences demonstrated high selectivity toward long-chain aldehydes, confirming their potential as receptor layers in peptide-based biosensors [20]. However, acknowledging the possibility of cross-reactivity and off-target interactions, a broader assessment was undertaken to evaluate the sensor’s responses to other chemical classes (Figures S10–S13). To this end, a comprehensive panel of 36 volatile organic compounds (VOCs) was selected, encompassing a diverse range of chemical groups including aromatic and aliphatic hydrocarbons, as well as oxygenated compounds such as aldehydes, alcohols, phenols, carboxylic acids, ethers, and furans (acetaldehyde, acetophenone, benzaldehyde, benzene, benzothiazole, butanal, butyric acid, cyclohexane, decanal, dimethyl sulfide, 2,2-dimethyldecane, 2,5-dimethylfuran, ethanol, heptanal, hexanal, 2-hydroxyacetaldehyde, 4-hydroxyhexanal, isoprene, nonanal, octanal, xylene, pentanal, phenol, propanol, propionaldehyde, propyl acetate, propylcyclohexane, styrene, soluene).

**Figure 3:**
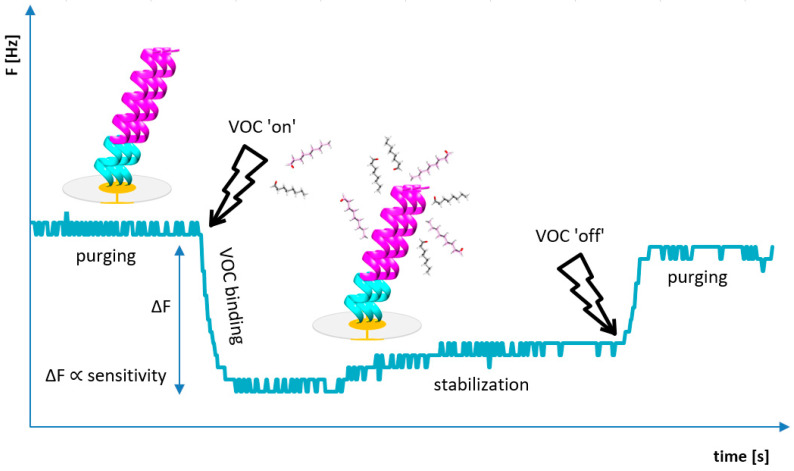
The individual stages of the typical single VOC analysis and resonant frequency responses (F). Initially, when the baseline for the peptide-based biosensor is established by flushing the system with air, the biosensor is exposed to a defined concentration of VOC. Upon exposure to the gas phase, the biosensor’s resonant frequency decreased progressively until a steady state was achieved, indicating maximal adsorption of the gas molecules onto the peptide layer. To complete the cycle, pure air was introduced, allowing the sensor signals to return to their original baseline values.

Measurements were carried out using the BioEnos Q6 platform developed by the Fahrenheit Union of Universities in Gdańsk (FarU), which allows real-time monitoring of frequency changes from six QCM sensors simultaneously. The sensors were mounted in a chamber constructed from polytetrafluoroethylene (PTFE), with a membrane pump placed downstream to ensure consistent gas flow. A three-way valve directed either the analyte gas or filtered air (via a purification unit) into the chamber, depending on the selected mode. A microcontroller governed the entire system, overseeing valve switching, signal acquisition from frequency counters, and controlling the pump speed by adjusting the supply voltage. Communication with a PC was facilitated through custom-developed “QCM enos” v6 software (FarU), which enabled system calibration, signal logging, and real-time visualization of frequency shifts. Prior to each measurement, a reference signal was acquired from an unmodified QCM sensor, which served as a baseline for further data interpretation. The experimental workflow followed a previously established measurement protocol (Figure 3). These signal changes correspond to the passage of sample and zero air through the QCM chamber. Measurement procedures followed a standardized protocol, and to ensure reliable interpretation, the first position was left empty (without a sensor). The appearance of a double plateau is attributed to flow interruption (elimination of advection), while the overshoot observed during desorption results from the high-flow purging that is used to accelerate sensor regeneration. Analytical output was defined as the frequency shift ∆F, which was calculated as the difference between the initial baseline frequency (F_0_) and the stabilized response frequency after analyte exposure F_R_, (∆F = F_0_ − F_R_).

## 3. Results

### 3.1. In Silico Results of Binding Affinity Between Peptide Salts and VOCs

The docking calculations revealed that long-chain aldehydes, both octanal and nonanal, bind within the −5.0 to −5.8 kcal·mol^−1^ energy range and to all receptor variants. For the OBPP4 without counter-ions, binding affinities were −5.2 to −5.6 kcal·mol^−1^, while the presence of chloride ions yielded very similar values (−5.2 to −5.5 kcal·mol^−1^). Slightly weaker interactions were observed in acetate-containing systems (−5.0 to −5.1 kcal·mol^−1^). On the other hand, the OBPP4 GSGSGS peptide displayed a comparable profile, yet had affinities ranging from −5.4 to −5.8 kcal·mol^−1^ in the chloride system, indicating slightly improved binding relative to the parent peptide. These results suggest that the detailed procedure of peptide deposition, particularly the presence and nature of counter-ions, may modulate the binding environment, though they do not fundamentally alter the receptors’ general preference towards long-chain aldehydes.

These results indicate a weak or moderate and reversible association. However, within an actual peptide monolayer, VOCs are not expected to bind to a single, well-defined deep pocket; instead, they likely interact with multiple “adsorption niches”. Accordingly, the occurrence of several docked poses with comparable scores may reflect partial nonspecific adsorption, which, in the experiment, contributes to the QCM mass-uptake signal. In this context, selectivity is expected to arise primarily from hydrophobic association and local chemical complementarity within the recognition layer (e.g., van der Waals contacts and the potential to participate in reversible Schiff base interactions, the latter of which is not captured by classical docking calculations).

In contrast, counter-ion exchange only had a minor effect on docking scores but substantially affected peptide monolayer packing density. Therefore, the enhanced experimental sensitivity most likely results from changes in sorption capacity and in the accessibility of adsorption sites at the peptide layer surface. More pronounced effects of counter-ions were observed in the analysis of the packing density. For the OBPP4 monolayer, the index increased from 0.68 in the absence of counter-ions to 0.77 with chloride, while acetate yielded an intermediate value of 0.73. In contrast, the GSGSGS-modified peptide showed more stable behavior, with indices between 0.72 and 0.74 irrespective of the counter-ion type. This indicates that the GSGSGS linker already stabilizes the organization of the peptide monolayer, making it less sensitive to the ionic environment. Representative models of both sensors with chloride ions are shown in Figure 2.

Taken together, these results point to two complementary conclusions. First, aldehyde-binding affinity remains largely unaffected by the choice of counter-ions, suggesting that recognition of long-chain aldehydes is an intrinsic property of the peptide motifs. Second, counter-ions can significantly influence the packing of peptide monolayers, with chloride ions leading to the highest packing densities in the OBPP4 system. Importantly, the presence of the GSGSGS linker significantly eliminates this variability, yielding receptor layers of uniform compactness and stability. These findings highlight the dual role of counter-ions and linkers in shaping the performance of peptide-based sensors and underline the importance of considering the electrostatic environment in biosensor design.

### 3.2. Peptides Deposition Efficiency onto Piezoelectric Transducers

**Figure 4:**
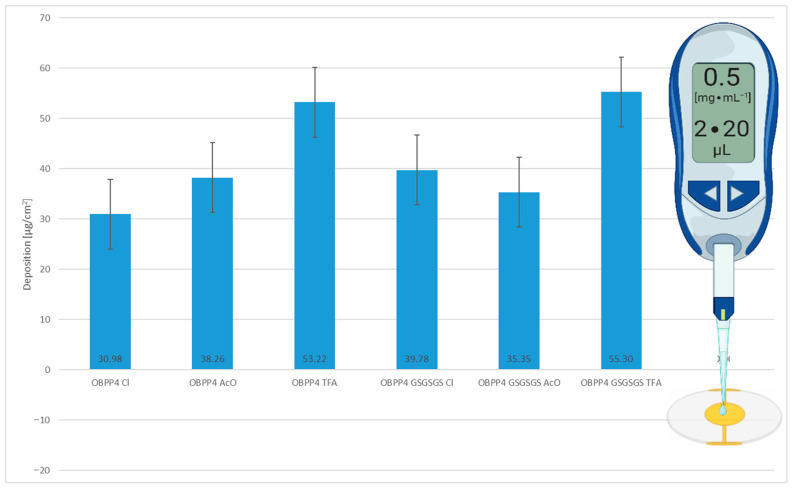
Mass loading of OBP-derived peptides on QCM sensors.

Peptide deposition on each biosensor was quantified by measuring frequency shifts before and after immobilization, as shown in Figure 4. The amount of mass that was loaded onto the sensor surface was determined using the Sauerbrey equation, following a previously validated protocol. As highlighted in earlier studies [35], the density and homogeneity of the biorecognition layer significantly impact both receptor–ligand binding kinetics and the resulting affinity. To ensure reliable comparisons across different sensors, the deposition parameters were carefully optimized to produce uniform surface coverage. Each deposition process was carried out according to a previously optimized method [35]. This approach reduced the variability related to peptide loading, thus improving the accuracy of VOC selectivity evaluations.

The peptide coverage that was achieved using a total volume of 40 µL of a 0.5 mg·mL^−1^ solution ranged from 30.98 ± 2.84 µg∙cm^−2^ for OBPP4 Cl^−^ to 55.29 ± 8.08 µg∙cm^−2^ for OBPP4 GSGSGS TFA. Intermediate deposition values were observed for OBPP4 AcO^−^ (38.26 ± 4.69 µg∙cm^−2^), OBPP4 TFA^−^ (53.22 ± 17.75 µg∙cm^−2^), OBPP4 GSGSGS Cl^−^ (39.77 ± 6.12 µg∙cm^−2^), and OBPP4 GSGSGS AcO^−^ (35.34 ± 8.24 µg∙cm^−2^). Notably, although OBPP4 GSGSGS Cl^−^ showed one of the lowest deposition amounts among the tested variants, its sensing performance toward long-chain aldehydes was among the most promising (Figure 4). This suggests that sensor response is influenced not only by deposition mass but also by the chemical nature and conformation of the immobilized peptide layer and the influence of counter-ions, which is an important insight for the future design of peptide-based gas sensors.

### 3.3. Detection of Aldehydes in Gas Phase

As demonstrated in previous studies [18,19], the biosensor incorporating the OBPP4 peptide as the active element exhibited high affinity toward long-chain aldehydes. In this study, peptide-functionalized biosensors were evaluated using a set of aldehydes that are listed in Table 2 as reference compounds. The adsorption kinetics were comparable across all tested sensors, showing an initial rapid frequency drop upon gas injection, followed by a slower stabilization phase leading to a steady state. The measurement protocol is schematically illustrated in Figure 3, including the operation of the pump regulating gas flow. Total measurement duration depended on signal stabilization both before and after analyte introduction, as well as the desorption rate. Each measurement cycle included baseline stabilization, analyte introduction and adsorption, response stabilization, and sensor regeneration. Biosensors coated with OBPP4 GSGSGS AcO^−^ showed responses very similar to or lower than those obtained with OBPP4 AcO^−^. For this reason, the results for the OBPP4 GSGSGS AcO^−^ sensor have been omitted from the main figures.

Biosensors functionalized with peptides from two distinct design strategies were tested against a panel of aldehydesand compounds from other chemical groups, as shown in Table 1 (Table S1). Characteristic plateau responses following the adsorption equilibrium were observed. Prior to these measurements, sensor drift was assessed using zero air (the background matrix for VOC preparation), and the drift did not exceed 2–3% at 43.5% relative humidity (Figure S2). The representative sensorgrams for uncoated and peptide-coated QCM electrodes that were recorded via the electronic nose software are provided in the Supplementary Materials. Additionally, response values for aldehydes that elicited no measurable interaction are also presented in the Supplementary Materials (Figure S9).

**Figure 5:**
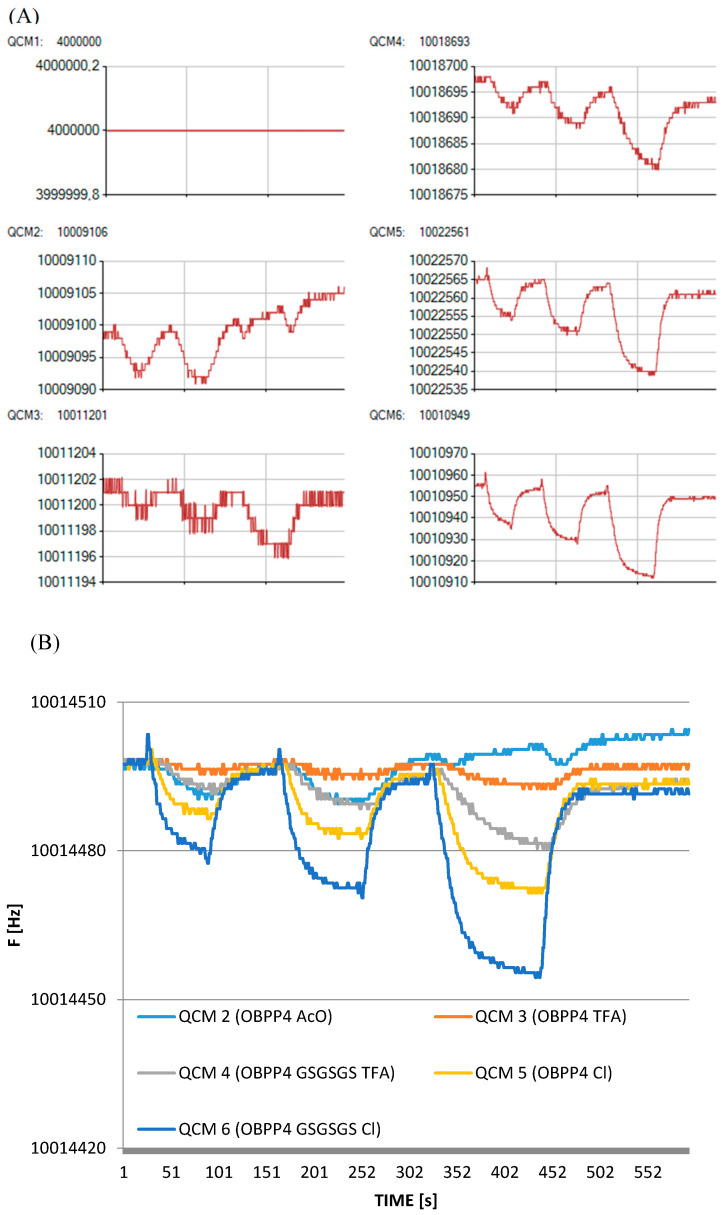
(**A**) Biosensors’ responses to nonanal at three concentrations: 27.97, 139.86, and 279.72 ppm, with the graphs directly exported from the software. QCM 1: empty slot, QCM 2: OBPP4 AcO^−^, QCM 3: OBPP4 TFA^−^, QCM 4: OBPP4 GSGSGS TFA^−^, QCM 5: OBPP4 Cl^−^, and QCM 6: OBPP4 GSGSGS Cl. (**B**) Normalized sensors’ responses to nonanal in three concentrations: 27.97, 139.86, and 279.72 ppm. QCM 1: empty slot (not shown), QCM 2: OBPP4 AcO^−^, QCM 3: OBPP4 TFA^−^, QCM 4: OBPP4 GSGSGS TFA^−^, QCM 5: OBPP4 Cl^−^, and QCM 6: OBPP4 GSGSGS Cl^−^.

**Figure 6:**
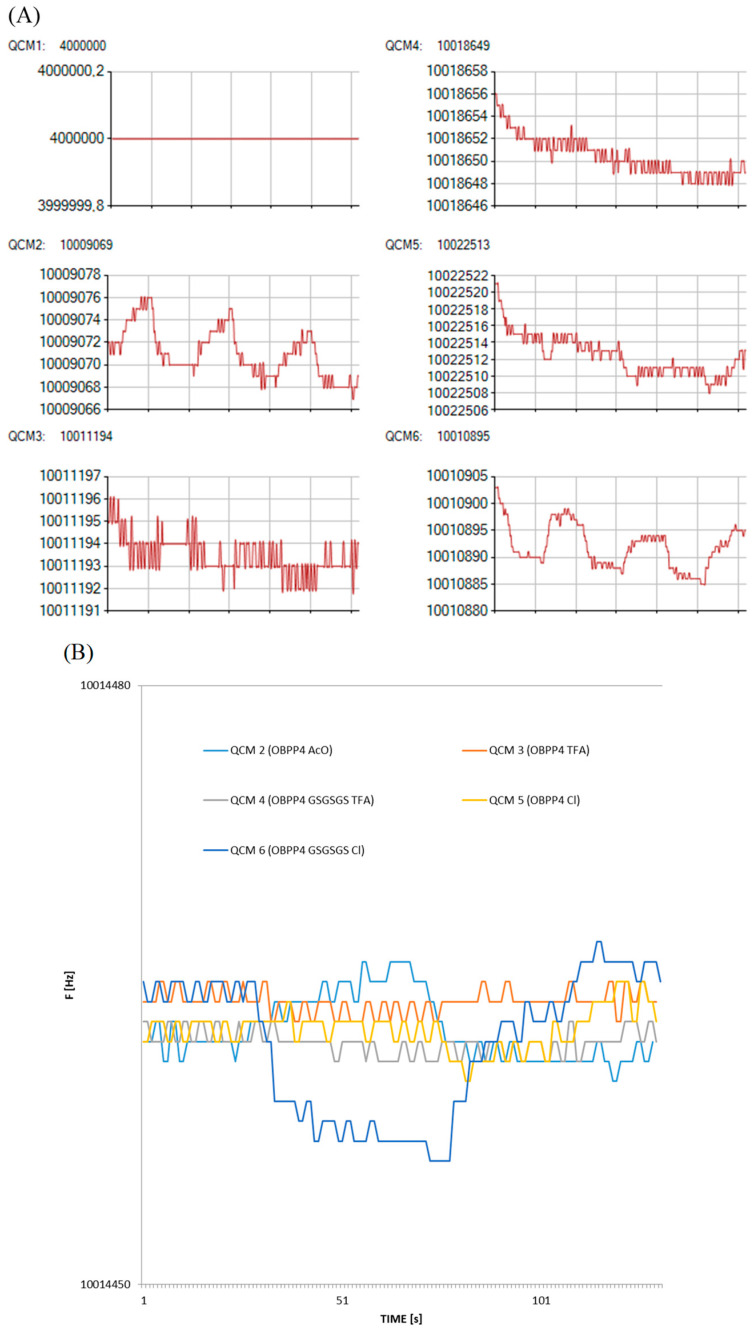
(**A**) The responses of biosensors to nonanal at a concentration of 5.59 ppm that were performed in three repetitions. The plot is directly exported from Bio-enose software (version 2.0). QCM 1: empty slot, QCM 2: OBPP4 AcO^−^, QCM 3: OBPP4 TFA^−^, QCM 4: OBPP4 GSGSGS TFA^−^, QCM 5: OBPP4 Cl^−^, and QCM 6: OBPP4 GSGSGS Cl^−^. (**B**) Response for nonanal at a concentration of 5.59 ppm, observed for the peptide OBPP4 GSGSGS Cl^−^.

Despite its relatively modest peptide loading (39.78 ± 6.12 µg·cm^−2^), OBPP4 GSGSGS Cl^−^ showed superior response magnitudes to long-chain aldehydes (nonanal, heptanal, and octanal), highlighting its strong binding affinity. Frequency shifts (ΔF) were recorded at various analyte concentrations to assess sensor sensitivity. The strongest responses were observed for nonanal and octanal, where OBPP4 GSGSGS Cl^−^ exhibited a ΔF up to 62 Hz at 279.72 ppm and 65 Hz at 308.06 ppm, respectively. The detection limits varied depending on the peptide and VOC tested, with responses as low as 2–5 Hz for sub-10 ppm concentrations of aldehydes. In contrast, sensor responses to aromatic hydrocarbons (e.g., toluene and ethylbenzene), alcohols (ethanol and propanol), and sulfur-containing compounds (DMS) remained below 5 Hz across all sensors, confirming high selectivity for aldehydes from previous studies [19]. Notably, only the biosensor modified with the OBPP4 GSGSGS peptide in the chloride salt form (Cl^−^) exhibited a measurable response at the lowest tested concentration of nonanal (5.59 ppm), yielding an average frequency shift of 12 Hz (Figure 5). To verify the biosensor responses at the lowest nonanal concentration used in this study, additional measurements were performed at 5.59 ppm in three independent repetitions (Figure 6A). At this concentration, the responses of OBPP4 AcO^−^, OBPP4 TFA^−^ and OBPP4 GSGSGS TFA^−^ remained within the baseline noise. In contrast, the OBPP4 Cl^−^ sensor showed distinguishable signals, yielding small but reproducible frequency shifts, which represent the most stable responses. A closer inspection of the raw sensorgrams for OBPP4 GSGSGS Cl^−^ (Figure 6B) reveals well-defined adsorption–desorption cycles with good repeatability across three runs, and a consistent *plateau* region that can be distinguished from baseline fluctuations. To validate sensor long-term performance, we assessed the stability through repeated exposures to nonanal (27.97 ppm) and octanal (30.81 ppm) over 14 days. OBPP4 GSGSGS Cl^−^ maintains > 90% of their initial response after 2 weeks of repeated exposures, demonstrating good operational stability and reversibility (Figure S15).

In contrast, no significant response was recorded for any of the biosensors when exposed to other aldehydes at comparable concentration levels. Complete response profiles and dynamic ranges are summarized in Supplementary Table S1. Based on the performed measurements, basic metrological parameters of the tested sensors were evaluated (Table 2).

**Figure 7:**
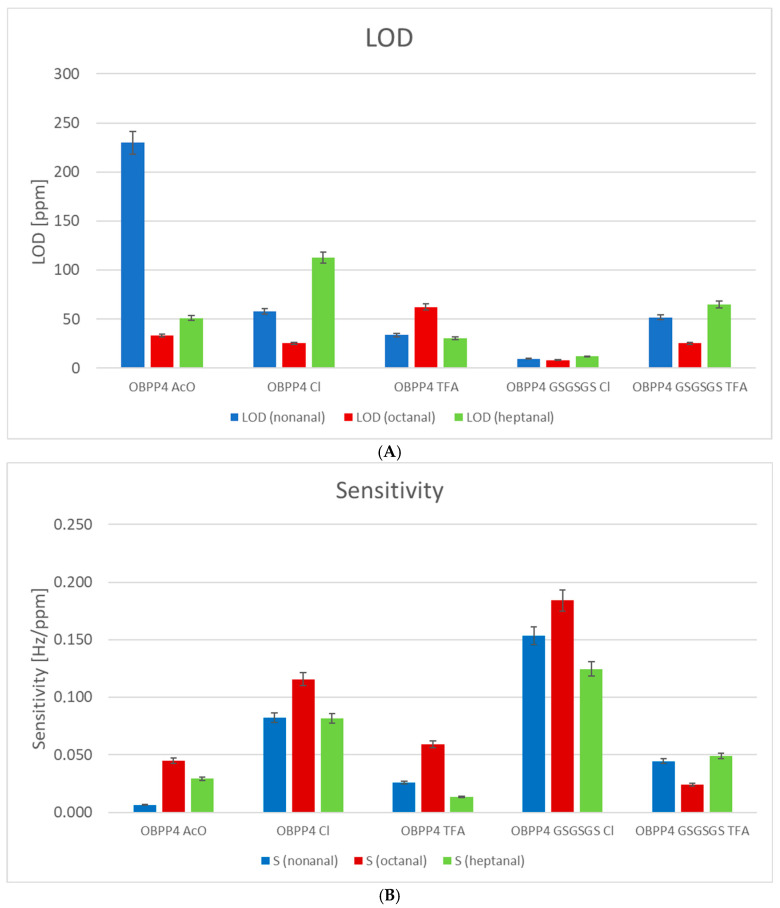
Parameters of the biosensors’ performance: (**A**) the LOD of nonanal (LOD 9), octanal (LOD 8) and heptanal (LOD 7) and (**B**) the sensitivity to nonanal (S 9), octanal (S 8), and heptanal (S 7).

Table 2 presents a comparative summary of key metrological parameters: sensitivity (*S*), limit of detection (LOD), and coefficient of determination (R^2^) for OBP-based sensors that were tested against six volatile organic compounds (VOCs): nonanal, octanal, heptanal, acetaldehyde, benzaldehyde, and formaldehyde. The limit of detection was estimated based on the static characteristic using Formula (2):

(2) $$LOD=\frac{3.3\cdot S_{Y}}{S}$$

where $S_{Y}$ is the standard deviation of the blank (zero-air) signal and S is the sensor sensitivity (i.e., the slope of the calibration curve expressed in Hz/ppm). The baseline exhibited very low noise, with $S_{Y}$ consistently in the range of 0.4–0.6 Hz, ensuring reliable and reproducible LOD estimation across all biosensors. This methodology follows the standard IUPAC recommendation for LOD determination in sensor measurements. The results indicate considerable variability in sensor performance depending on both the sensor type and the analyte. The most promising responses, characterized by high sensitivity, low LOD values, and R^2^ values approaching unity, were observed primarily for OBPP4 Cl^−^ and OBPP4 GSGSGS Cl^−^, particularly in response to nonanal, octanal, benzaldehyde, and formaldehyde. In contrast, no significant frequency changes were detected for any of the sensors in response to acetaldehyde, indicating a lack of detectable interaction under the tested conditions (Figure S5). Similarly, no noticeable response was recorded for other groups of VOCs, i.e., alcohols, phenols, carboxylic acids, ethers, and furans. These findings highlight the selectivity and performance variability among OBP-based sensors and point to specific candidates for further development in VOC detection applications. For better visualization, the LOD and sensitivity data for heptanal, octanal, and nonanal are presented in Figure 7.

Among the evaluated sensors, OBPP4 GSGSGS Cl^−^ and OBPP4 Cl^−^ exhibited the most promising performance in detecting long-chain aldehydes, including nonanal, octanal, and heptanal. For nonanal, OBPP4 GSGSGS Cl^−^ achieved the highest sensitivity (S = 0.153 Hz/ppm), the lowest detection limit (LOD = 9.8 ppm), and an excellent coefficient of determination (R^2^ = 0.961), while OBPP4 Cl^−^ also performed well, with S = 0.082 Hz/ppm, LOD = 18.2 ppm, and R^2^ = 0.872. In the case of octanal, OBPP4 GSGSGS Cl^−^ again showed a superior sensitivity (S = 0.184 Hz/ppm) and a lower LOD (8.1 ppm) compared to OBPP4 Cl^−^ (S = 0.116 Hz/ppm, LOD = 12.9 ppm), though both sensors achieved high R^2^ values (0.925 and 0.858, respectively). Nevertheless, other peptides did not provide high sensitivity, and one that should be borne in mind is that the purity of the peptide acetates was noticeably lower than that of the remaining peptides. Therefore, the results for peptide acetates are not easy to evaluate and compare with those of the other peptides. For heptanal, the performance was more comparable: OBPP4 Cl^−^ reached S = 0.125 Hz/ppm, LOD = 12.0 ppm, and R^2^ = 0.798, while OBPP4 GSGSGS Cl^−^ exhibited similar sensitivity (S = 0.082 Hz/ppm), slightly higher LOD (18.3 ppm), and R^2^ = 0.781. Overall, although OBPP4 GSGSGS Cl^−^ recorded the highest individual metrics, OBPP4 Cl^−^ demonstrated more consistent and well-balanced performance across all three aldehydes, suggesting that both peptides have strong potential as broadly effective recognition layers in biosensors for long-chain aliphatic aldehyde detection.

**Figure 8:**
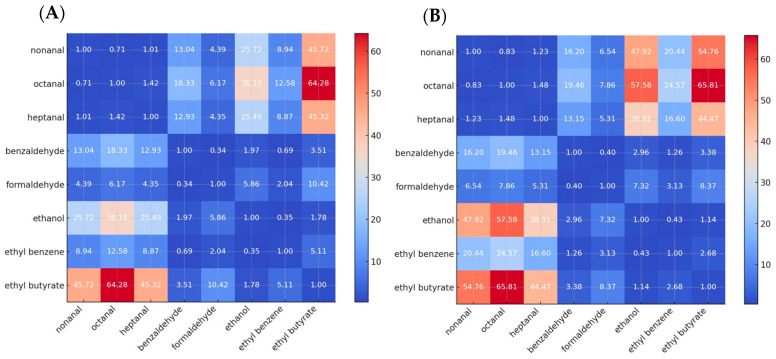
Selectivity maps for the two best-performing biosensors tested against various VOCs: (**A**) OBPP4 Cl^−^ sensor and (**B**) OBPP4 GSGSGS Cl^−^ sensor.

For the two most promising sensors, OBPP4 GSGSGS Cl^−^ and OBPP4 Cl^−^, selectivity coefficients towards the tested volatile compounds were calculated based on Equation (3). The results are presented in Figure 7.

(3) $$K_{AB}=\frac{S_{A}}{S_{B}}$$

where *K_AB_* denotes the sensor’s selectivity coefficient, *S_A_* represents the sensor’s sensitivity to analyte *A*, and *S_B_* corresponds to the sensitivity to analyte *B*. The selectivity of the two best-performing biosensors (OBPP4 Cl^−^ and OBPP4 GSGSGS Cl^−^) was evaluated by calculating the selectivity coefficients *K_AB_*, which were defined as the ratio of sensitivities (Sa/SB) that were obtained independently from calibration curves for each VOC. This approach does not involve simultaneous exposure to two gases; instead, it quantifies how much more strongly the sensor responds to a target aldehyde compared with another compound. The aim of this experiment was to demonstrate that both sensors exhibit a very high selectivity toward long-chain aldehydes (nonanal, octanal, and heptanal), while their responses to short-chain aldehydes or other VOC groups remain very low or undetectable. The resulting selectivity maps (Figure 8) visualize this discrimination capability across all tested VOCs. High values indicate strong selectivity of the peptide-based biosensors for long-chain aldehydes (nonanal, octanal, and heptanal) over other tested VOCs.

The selectivity heatmaps (Figure 8) indicate that both OBPP4 GSGSGS Cl^−^ and OBPP4 Cl^−^ sensors exhibit markedly higher selectivity toward long-chain aliphatic aldehydes, nonanal, octanal (Figure S6), and heptanal (Figure S8), when compared to short-chain aldehydes and other volatile organic compounds (VOCs). For instance, the OBPP4 GSGSGS Cl^−^ sensor showed a selectivity of 45.7 for nonanal over ethyl butyrate, 36.2 for octanal over ethanol, and 12.9 for heptanal over benzaldehyde. Similarly, the OBPP4 Cl^−^ sensor reached even higher values, such as 65.8 for octanal over ethyl butyrate, 47.9 for nonanal over ethanol, and 13.2 for heptanal over benzaldehyde. These consistently elevated selectivity ratios, often exceeding 40 when comparing long-chain aldehydes to alcohols or esters, highlight the strong discrimination capability of both sensors. While OBPP4 Cl^−^ demonstrates slightly higher absolute selectivity values, OBPP4 GSGSGS Cl^−^ provides a more uniform response profile, suggesting better overall robustness for long-chain aldehyde detection in complex VOC mixtures. The estimated LOD in the chloride-based peptide relative to the previous TFA^−^ based reflects methodological differences in the calibration range selection. The previous linker optimization study [20] employed a focused concentration range calibration, while the current work utilizes a more comprehensive calibration range, 5.59–279.72 ppm, encompassing the full sensor dynamic range.

## 4. Discussion

The results obtained confirm the strong and selective interaction of OBPP4-based QCM sensors with long-chain aldehydes. Among the tested receptors, OBPP4 GSGSGS Cl^−^ exhibited the highest affinity, particularly towards nonanal and octanal, despite moderate surface loading. This suggests that sensor performance is influenced not solely by peptide quantity but also by molecular conformation and the accessibility of binding residues. Aldehyde binding energies are broadly similar across “no counterion” vs. chloride, with acetate slightly weaker, but packing density changes were more pronounced; the improved sensitivity likely arises from monolayer organization and site accessibility rather than a large change in intrinsic peptide–aldehyde interaction energy. The strong selectivity of the OBPP4 Cl^−^ and OBPP4 GSGSGS Cl^−^ sensors toward the long-chain aldehydes arises from the combined influence of molecular recognition and monolayer organization. Long-chain aldehydes interact favorably with the hydrophobic regions of the peptide layer, and their chain length enables stable accommodation within the adsorption grooves formed by peptide packing. In contrast, short-chain aldehydes and other VOC classes lack sufficient hydrophobic interaction area or steric complementarity, resulting in negligible responses. This behavior correlates with our MD-derived packing density results, which show that chloride counter-ions promote a more compact and homogeneous monolayer, enhancing analyte accessibility and interaction strength. The GSGSGS linker further stabilizes peptide orientation, reducing structural variability and contributing to more uniform binding sites. These structural effects provide an explanation for the increased sensitivity and selectivity observed experimentally. Together, these findings confirm that the high selectivity of the peptide-based sensors is not incidental but results from the combined physicochemical effects of the peptide sequence design, counter-ion choice, and monolayer organization.

## 5. Conclusions

The use of the GSGSGS linker enhances flexibility and spatial orientation of the binding site, improving analyte accommodation. The presence of different counter-ions (Cl^−^, AcO^−^, TFA^−^) impacted both the deposition efficiency and sensor response. Peptides with TFA^−^ showed the highest deposition levels but not necessarily the best performance, which may be attributed to altered peptide charge states or aggregation during immobilization. The original peptide OBPP4 exhibited lower sensitivity to long-chain aldehydes than the peptide with the GSGSGS linker, which confirms our previous reports [20]. Counter-ion exchange offers a practical post-synthesis optimization, as it is simple, cost-effective, and applicable to existing TFA-peptides. The linker study established peptide design principles (linear correlation between linker length and sensitivity), while this work reveals electrostatic fine-tuning as an enhancement strategy. Future optimization should combine both approaches, using linker-modified peptides in chloride form, potentially achieving synergistic effects beyond either method alone. This study provides evidence that the LOD for long-chain aldehydes, particularly nonanal and octanal, can be enhanced by substituting the counter-ion with chloride. This effect is more pronounced in OBPP4 compared to OBPP4 GSGSGS. Furthermore, biosensors modified with peptide chlorides exhibit significantly greater sensitivity to these aldehydes. The selectivity pattern observed across VOCs confirms that peptide design and deposition play a critical role in shaping sensor recognition properties. The minimal responses toward alcohol and aromatic hydrocarbons highlight the biosensor’s performance for aldehyde-specific detection in complex breath matrices. Given the strong responses to disease-related aldehydes and the low cross-sensitivity to other VOCs, OBPP4 GSGSGS Cl^−^, in particular, emerges as a promising candidate for further development of diagnostic biosensor development. These findings highlight the strong influence of the counter-ion on biosensor response, offering a valuable avenue for optimizing peptide- and protein-based biosensors.

## Figures and Tables

**Table 1:** The concentrations of aldehydes [ppm] that were prepared in Tedlar bags before analysis.

VOCs	C_1_ (1 µL/25 L)	C_2_ (1 µL/5 L)	C_3_ (5 µL/5 L)	C_4_ (10 µL/5 L)
Acetaldehyde	17.12	85.62	428.11	856.22
Benzaldehyde	9.43	47.15	652.81	471.56
Formaldehyde	26.11	130.56	235.73	471.46
Nonanal	5.59	27.97	139.86	279.72
Octanal	6.16	30.81	154.03	308.06
Pentanal	9.04	45.21	226.07	452.14

**Table 2:** The basic metrological parameters of the tested biosensors against aldehydes.

Chemical Compound	Metrological Parameter	OBPP4 AcO^−^	OBPP4 TFA^−^	OBPP4 GSGSGS TFA^−^	OBPP4 Cl^−^	OBPP4 GSGSGS Cl^−^
Nonanal	S [Hz⋅ppm^−1^]	0.007	0.026	0.044	0.082	0.153
	LOD [ppm]	229.7	58.1	33.7	18.2	9.8
	R^2^ [-]	0.195	0.840	0.910	0.872	0.961
	S_n_	0.0002	0.0005	0.0008	0.0026	0.0038
Octanal	S [Hz⋅ppm^−1^]	0.045	0.059	0.024	0.116	0,184
	LOD [ppm]	33.3	25.4	62.2	12.9	8.1
	R^2^ [-]	0.732	0.874	0.697	0.858	0.925
	S_n_	0.0012	0.0011	0.0004	0.0037	0.0046
Heptanal	S [Hz⋅ppm^−1^]	0.029	0.013	0.049	0.082	0.125
	LOD [ppm]	51.2	113.0	30.4	18.3	12.0
	R^2^ [-]	0.819	0.721	0.830	0.781	0.798
	S_n_	0.0008	0.0002	0.0009	0.0026	0.0031
Acetaldehyde	S [Hz⋅ppm^−1^]	nr	nr	nr	nr	nr
	LOD [ppm]					
	R^2^ [-]					
	S_n_					
Benzaldehyde	S [Hz⋅ppm^−1^]	nr	0.004	nr	0.006	0.009
	LOD [ppm]		379.5		237.2	135.6
	R^2^ [-]		0.510		0.542	0.534
	S_n_		0.0001		0.0002	0.0002
Formaldehyde	S [Hz⋅ppm^−1^]	nr	nr	0.005	0.019	0.023
	LOD [ppm]			319.4	79.8	63.9
	R^2^ [-]			0.653	0.756	0.771
	S_n_			0.0001	0.0006	0.0006

S—sensitivity of the sensor, LOD—limit of detection, R^2^—coefficient of determination determined from the sensor’s static characteristic, S_n_—normalized sensitivity of the sensor [Hz⋅ppm^−1^⋅(μg⋅cm^−2^)^−1^], and nr—no response (no variation in the sensor’s frequency was detected).

## Data Availability

The raw data for this study is available in the ppm.gumed.edu.pl repository at https://ppm.gumed.edu.pl/info/researchdata/GUM6df43dfed46b46388b86fb5cfb83b107/ (accessed on 1 December 2025).

## Supplementary Materials

The following supporting information can be downloaded at: https://www.mdpi.com/article/10.3390/bios16030162/s1, Figure S1: Chromatogram obtained by HPLC analysis for the OBPPP4_GSGSGS peptide. Figure S2: Contact angle measurements for peptide-modified gold electrodes with and without oxygen plasma cleaning. Figure S3: Configuration of the electronic nose (EN) system used for gas detection and photograph of the experimental setup. Figure S4: Sensor drift measurement for the VOC preparation matrix showing minimal drift (±2 Hz). Figure S5: Biosensors’ responses to octanal at a concentration of 308.06 ppm. Figure S6: Biosensors’ responses to octanal at concentrations of 308.06, 154.03 and 30.81 ppm. Figure S7: Biosensors’ responses to nonanal at a concentration of 279.72 ppm. Figure S8: Biosensors’ responses to heptanal at a concentration of 344.22 ppm. Figure S9: Biosensors’ responses to acetaldehyde at a concentration of 856.22 ppm. Figure S10: Biosensors’ responses to ammonia at a concentration of 277.55 ppm. Figure S11: Biosensors’ responses to ethyl benzene at a concentration of 398.01 ppm. Figure S12: Biosensors’ responses to DMS at a concentration of 640.05 ppm. Figure S13: Biosensors’ responses to xylene at a concentration of 389.72 ppm. Figure S14: AFM images of biosensors: bare QCM sensor and peptide-based biosensor with GSGSGS linker and TFA^−^ counterion. Figure S15: Long-term stability of biosensors during repeated exposure to nonanal and octanal over 14 days. Table S1: Biosensors’ responses (ΔF [Hz]) to other gaseous compounds.
