# Supplementary material for: Enhancing the Detection of Long-Chain Aldehydes by Peptide-Based Biosensors Through Counter-Ion Exchange
Source: Biosensors (Basel). 2026 Mar 13;16(3):162. doi: 10.3390/bios16030162 (PMC13024638; doi:10.3390/bios16030162)
Supplement: Supplementary file 1 [file biosensors-16-00162-s001.zip › biosensors-4166661-supplementary.pdf]

SUPPLEMENTARY MATERIALS

Enhancing the Detection of Long-Chain Aldehydes by Peptide-Based Biosensors Through Counter-Ion Exchange

Tomasz Wasilewski [1,*], Damian Neubauer [1], Elisabete Fernandes [2], Rafał Kiejzik [1], Bartosz Szulczyński [3], Jacek Gębicki [3], Wojciech Kamysz [2] and Marek Wojciechowski [4]

[1] Department of Inorganic Chemistry, Faculty of Pharmacy, Medical University of Gdansk, Poland, Hallera 107, 80-416 Gdansk, Poland
[2] International Iberian Nanotechnology Laboratory, 4715-330 Braga, Portugal; elisabete.fernandes@inl.int (E.F.)
[3] Department of Process Engineering and Chemical Technology, Faculty of Chemistry, Gdansk University of Technology, 11/12 G. Narutowicza Street, 80-233 Gdansk, Poland
[4] Department of Pharmaceutical Technology and Biochemistry, Chemical Faculty, Gdańsk University of Technology, Narutowicza 11/12, 80-233 Gdańsk, Poland; marek.wojciechowski@pg.edu.pl
* Correspondence: tomasz.wasilewski@gumed.edu.pl

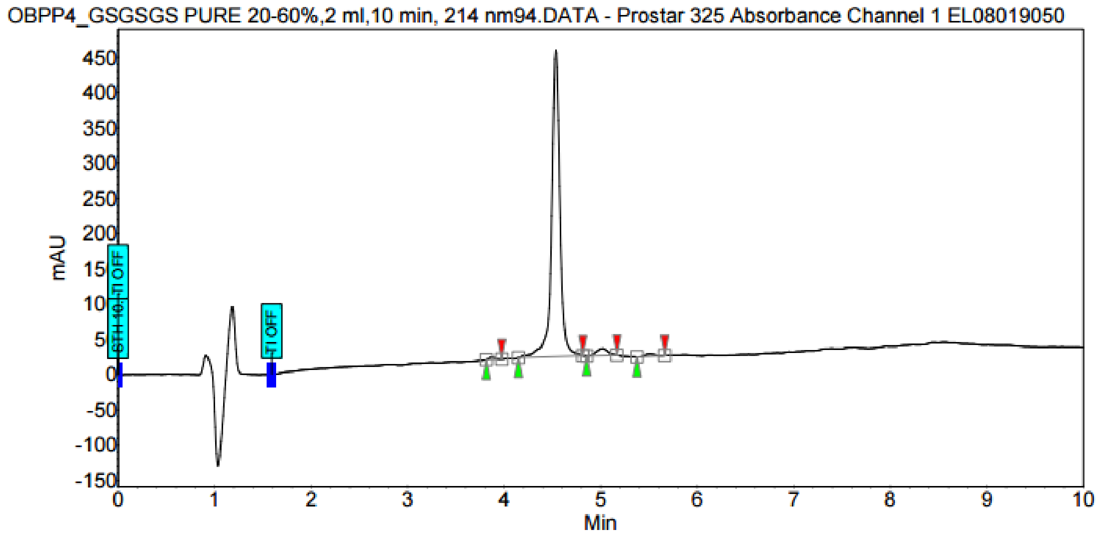

Figure S1. Chromatogram obtained by HPLC analysis for the OBPPP4_GSGSGS. The purifications of peptide amides were performed via reversed-phase high-performance liquid chromatography (RP-HPLC) using LP-chrom software. Analytical and preparative HPLC were conducted in a water/acetonitrile gradient on a Waters X-Bridge Prep C18 column. The purity of the peptides was confirmed to exceed 95% by HPLC analysis (Varian, Mulgrave, VIC, Australia).

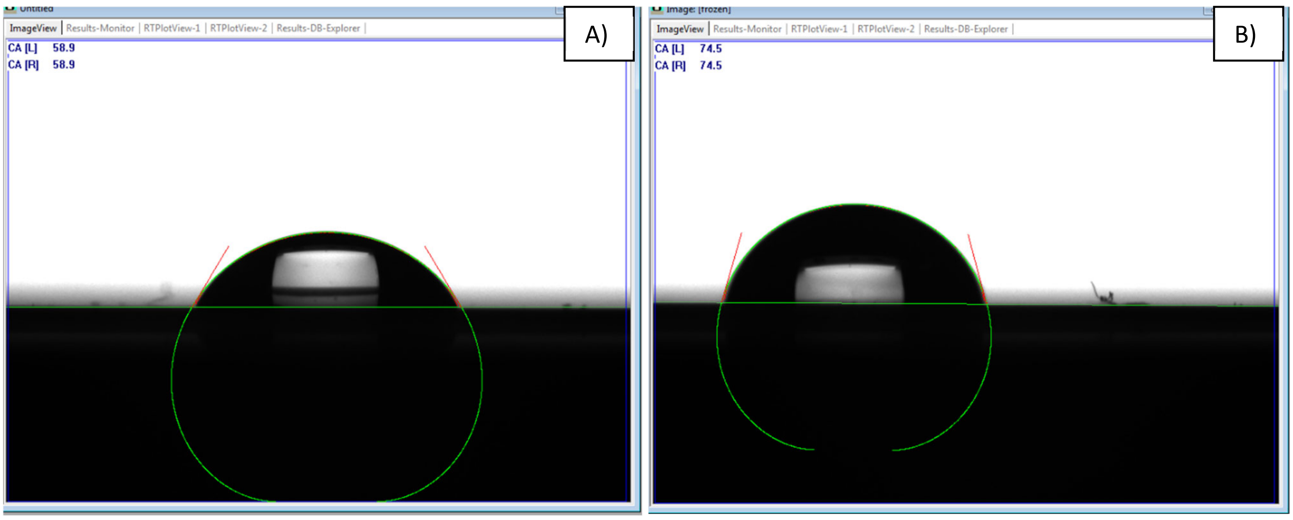

Figure S1. (A) Recorded contact angle (58.9°) for the sample after applying the peptide to a gold electrode cleaned with oxygen plasma. (B) Contact angle (74.5°) recorded for the gold QCM electrode after applying the peptide. The difference in the angle indicates a change in the hydrophobicity of the surface caused by oxygen plasma treatment.

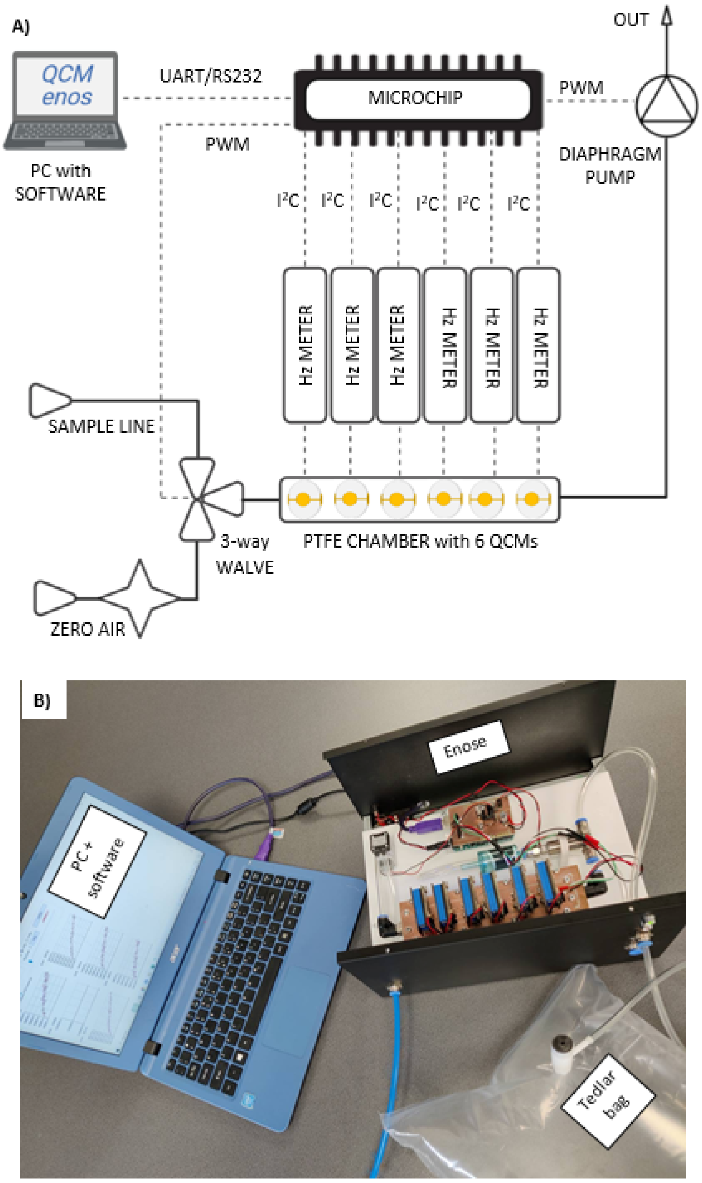

Figure S3. A) Schematic diagram illustrating the configuration of the EN system used for gas sample detection, and B) photograph showing the actual experimental setup.

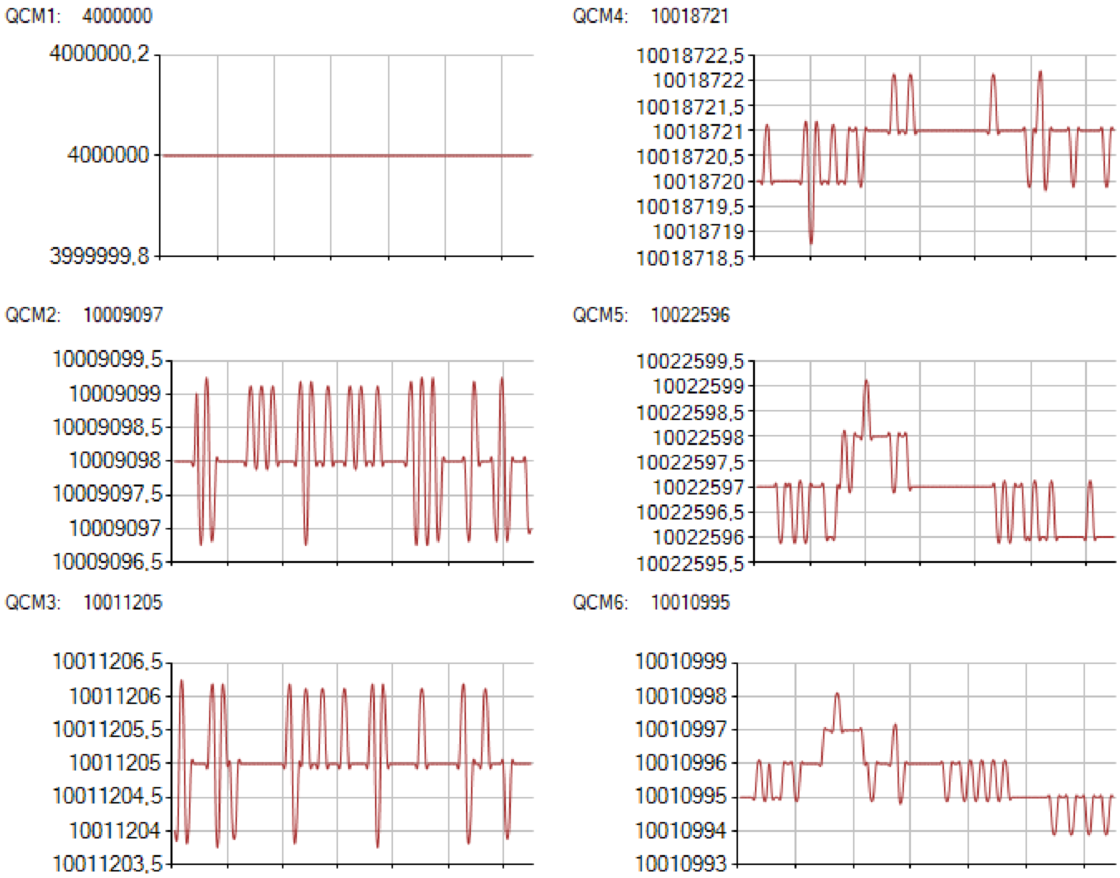

***Figure** S4. Measurement of the matrix in which the VOCs were prepared, where the minimal sensor drift of ±2 Hz is visible. QCM 1 → empty slot, QCM 2 → OBPP4 AcO-, QCM 3 → OBPP4 TFA-, QCM 4 → OBPP4 GSGSGS TFA-, QCM 5 → OBPP4 Cl-, and QCM 6 → OBPP4 GSGSGS Cl-.*

Table S1. Biosensors' responses (ΔF [Hz]) to other gaseous compounds, blank box - no response.

		QCM 2			QCM 3			QCM 4			QCM 5			QCM 6			QCM 1			
		**OBPP4 AcO-**			**OBPP4 GSGSGS TFA-**			**OBPP4 TFA-**			**OBPP4 Cl-**			**OBPP4 GSGSGS Cl-**			**OBPP4 GSGSGS AcO-**		Bare	
VOC	[ppm]	ΔF [Hz]																		
Acetaldehyde	856.22																			
	428.11																			
	85.62																			
	17.12																			
Benzaldehyde	652.81				4	4	2				6	4	6	10	6	8				
	471.56																			
	47.15																			
	9.43																			
Formaldehyde	471.46							3	2	1	10	8	6	10	12	8				
	235.73																			
	130.56																			
	26.11																			

Nonanal	279.72	5	5	3	8	10	6	15	11	11	25	23	32	51	62	54	10	12	12	
	139.86	5	6	2	4	5	4	10	8	7	15	11	20	30	35	30	3	2	2	
	27.97	4	4	3	2	2	4	3	1	2	10	5	11	17	22	16				
	5.59	4	2	0							2	2	2	10	12	14				
Octanal	308.06	20	11	15	20	22	18	6	8	10	35	37	39	61	62	65	25	28	30	
	154.03	15	6	10	15	14	12	6	6	8	12	15	35	40	33	35	11	12	11	
	30.81	5	4	8	9	8	5	5	0	4	5	8	11	25	20	18	3	3	3	
	6.16																			
Pentanal	452.14	10	8	5	3	5	3	15	22	11										
	226.07							2	2	3										
	45.21																			
	9.04																			
Heptanal	344.22	8	10	12	6	3	4	22	12	15	40	25	29	62	42	45	11	12	13	
	172.11	10	4	3	4	4	4	15	8	6	27	15	11	44	32	33	2	2	3	
	34.42	2	0	2	0	2	0	2	2	0	15	10	8	30	15	11				
	6.88													6	3	2				
DMS	640.05	4	4	3																
Ethanol	823.92	2	2	1				4	3	1	4	2	2	5	2	1				
Ethyl benzene	398.01										5	5	1	4	3	2				
Ethyl butyrate	362.39										2	0	0	3	0	0				
Toluene	298.1																			
Ammonia	277.55																			
Xylene	389.72																			

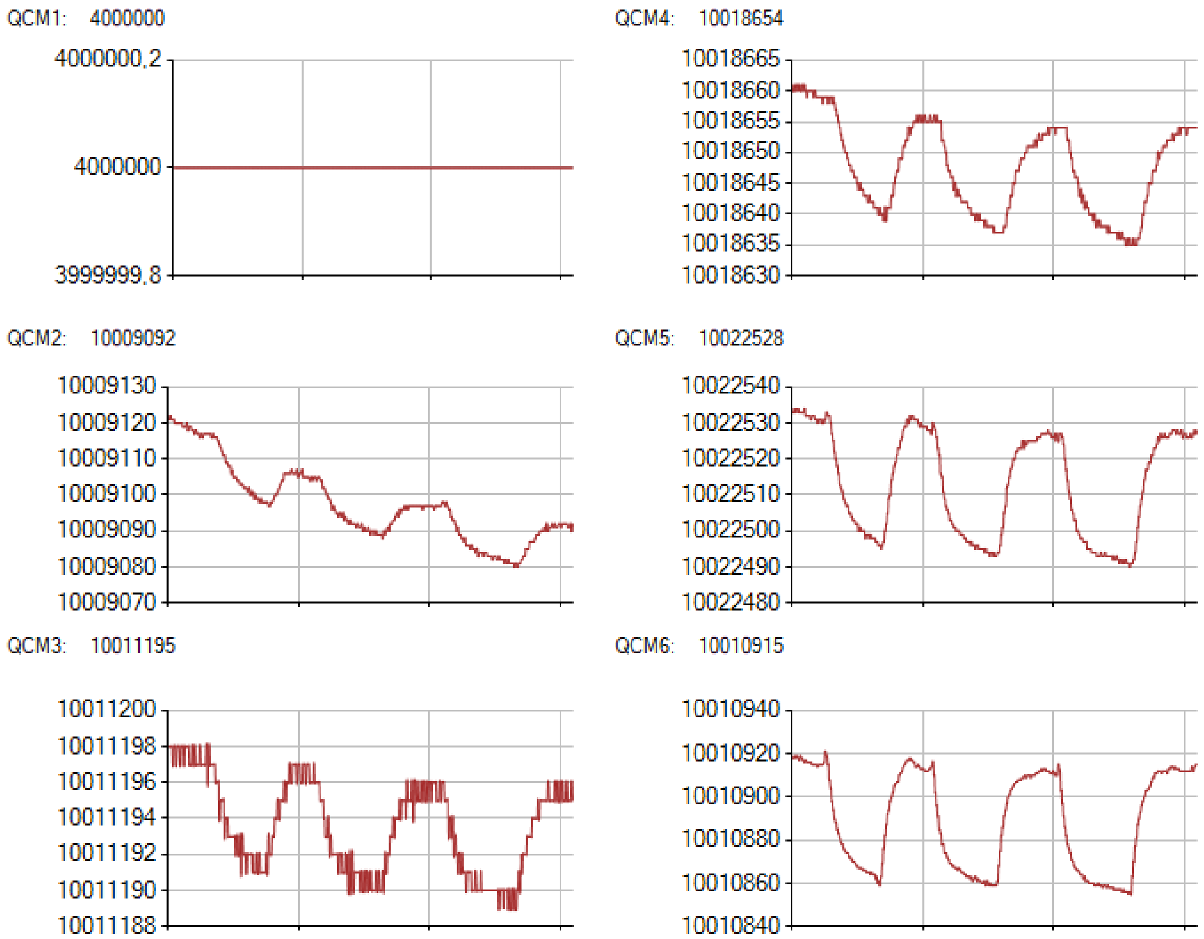

Figure S5. Biosensors' responses to octanal at the concentration of 308.06 ppm. The graphs were directly taken from the software. QCM 1 → empty slot, QCM 2 → OBPP4 AcO⁻, QCM 3 → OBPP4 TFA⁻, QCM 4 → OBPP4 GSGSGS TFA⁻, QCM 5 → OBPP4 Cl⁻, and QCM 6 → OBPP4 GSGSGS Cl⁻.

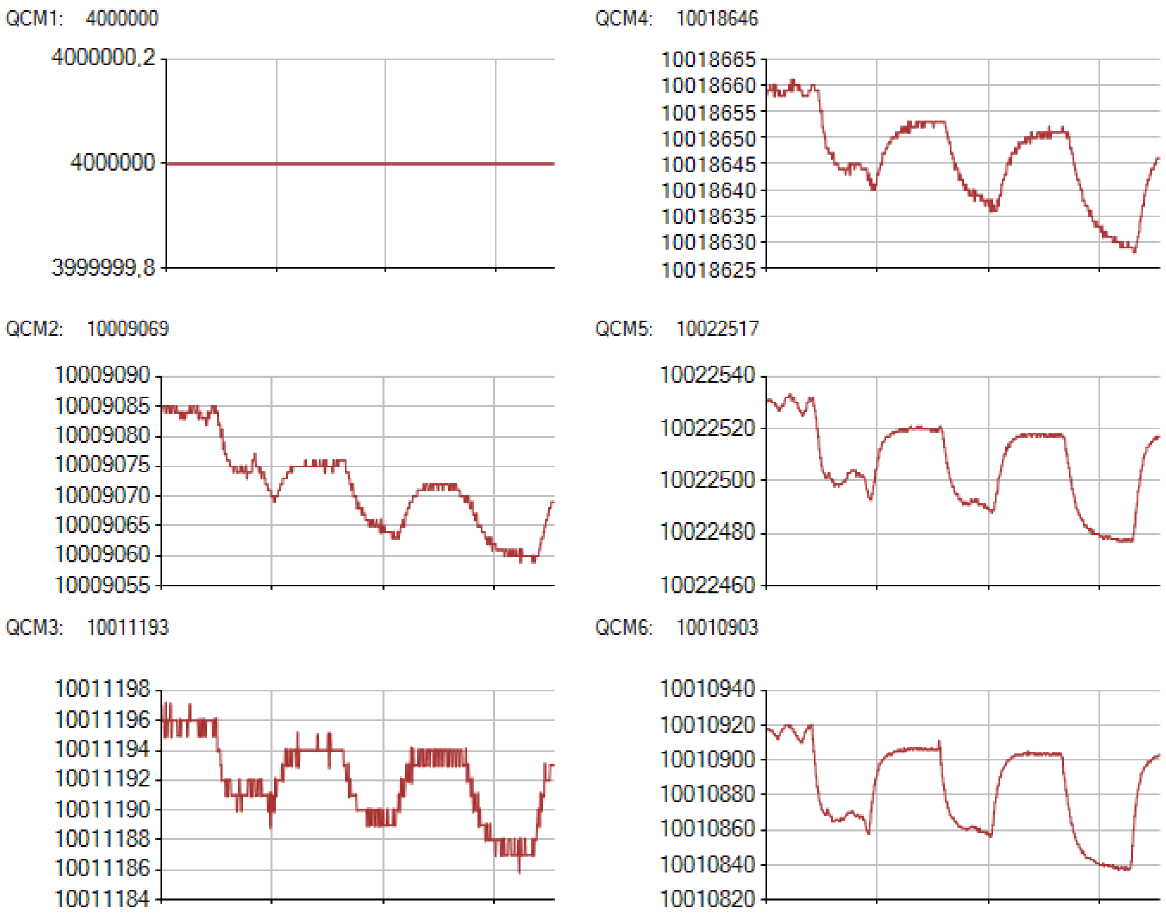

Figure S6. Biosensors' responses to octanal at the concentration of 308.06, 154.03 and 30.81 ppm. The graphs were directly taken from the software. QCM 1 → empty slot, QCM 2 → OBPP4 AcO⁻, QCM 3 → OBPP4 TFA⁻, QCM 4 → OBPP4 GSGSGS TFA⁻, QCM 5 → OBPP4 Cl⁻, and QCM 6 → OBPP4 GSGSGS Cl⁻

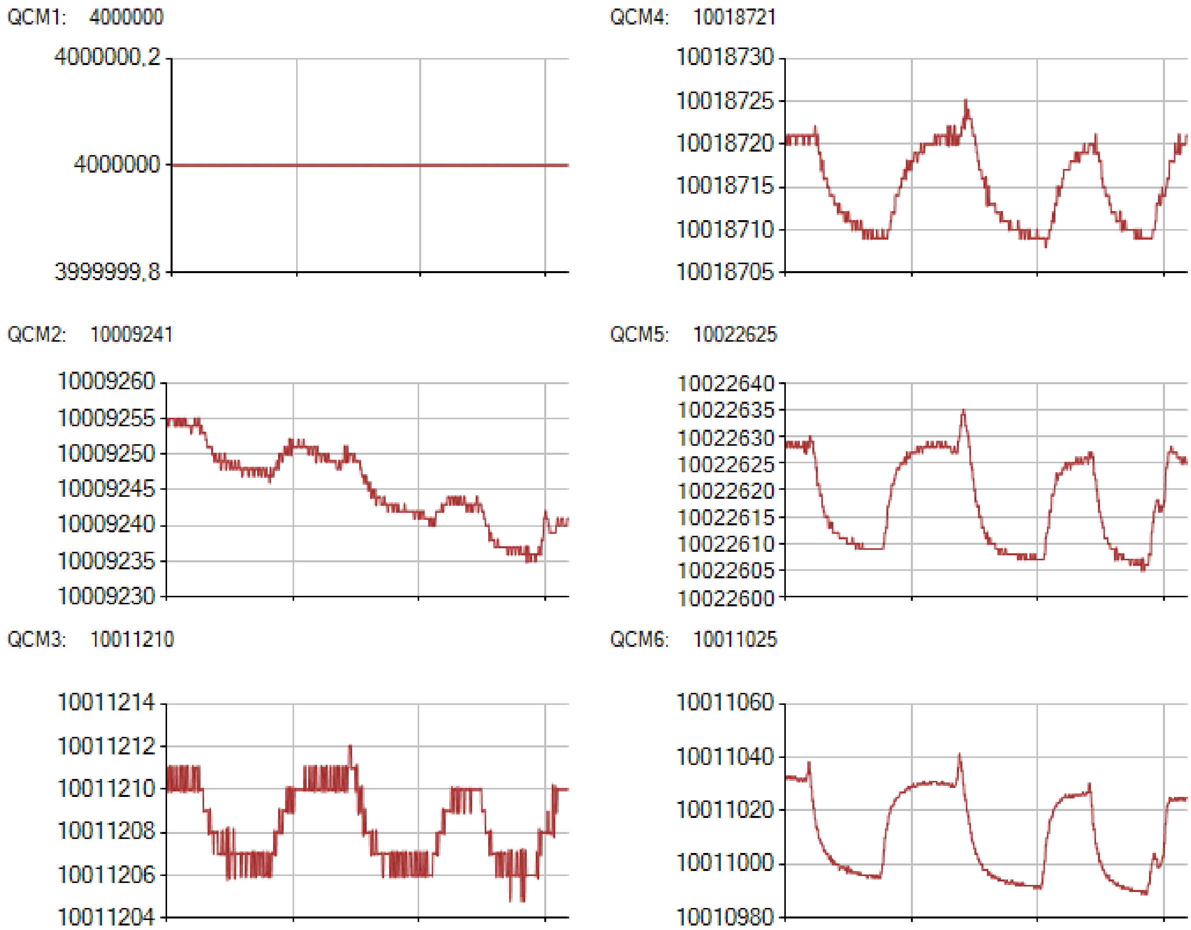

Figure S7. Biosensors' responses to nonanal at the concentration of 279.72 ppm. The graphs were directly taken from the software. QCM 1 → empty slot, QCM 2 → OBPP4 AcO$^-$, QCM 3 → OBPP4 TFA$^-$, QCM 4 → OBPP4 GSGSGS TFA$^-$, QCM 5 → OBPP4 Cl$^-$, and QCM 6 → OBPP4 GSGSGS Cl$^-$.

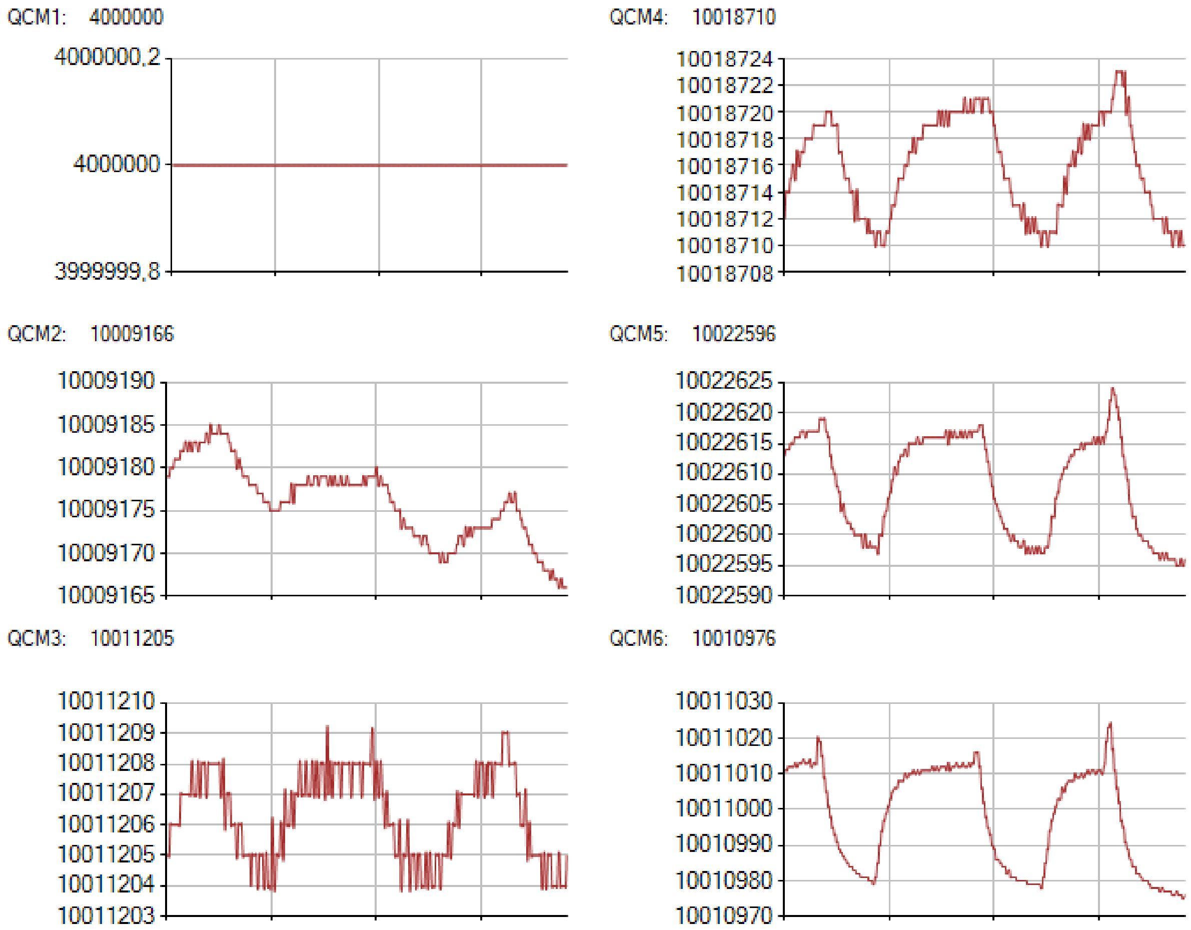

Figure S8. Biosensors' responses to heptanal at the concentration of 344.22 ppm. The graphs were directly taken from the software. QCM 1 → empty slot, QCM 2 → OBPP4 AcO⁻, QCM 3 → OBPP4 TFA⁻, QCM 4 → OBPP4 GSGSGS TFA⁻, QCM 5 → OBPP4 Cl⁻, and QCM 6 → OBPP4 GSGSGS Cl⁻.

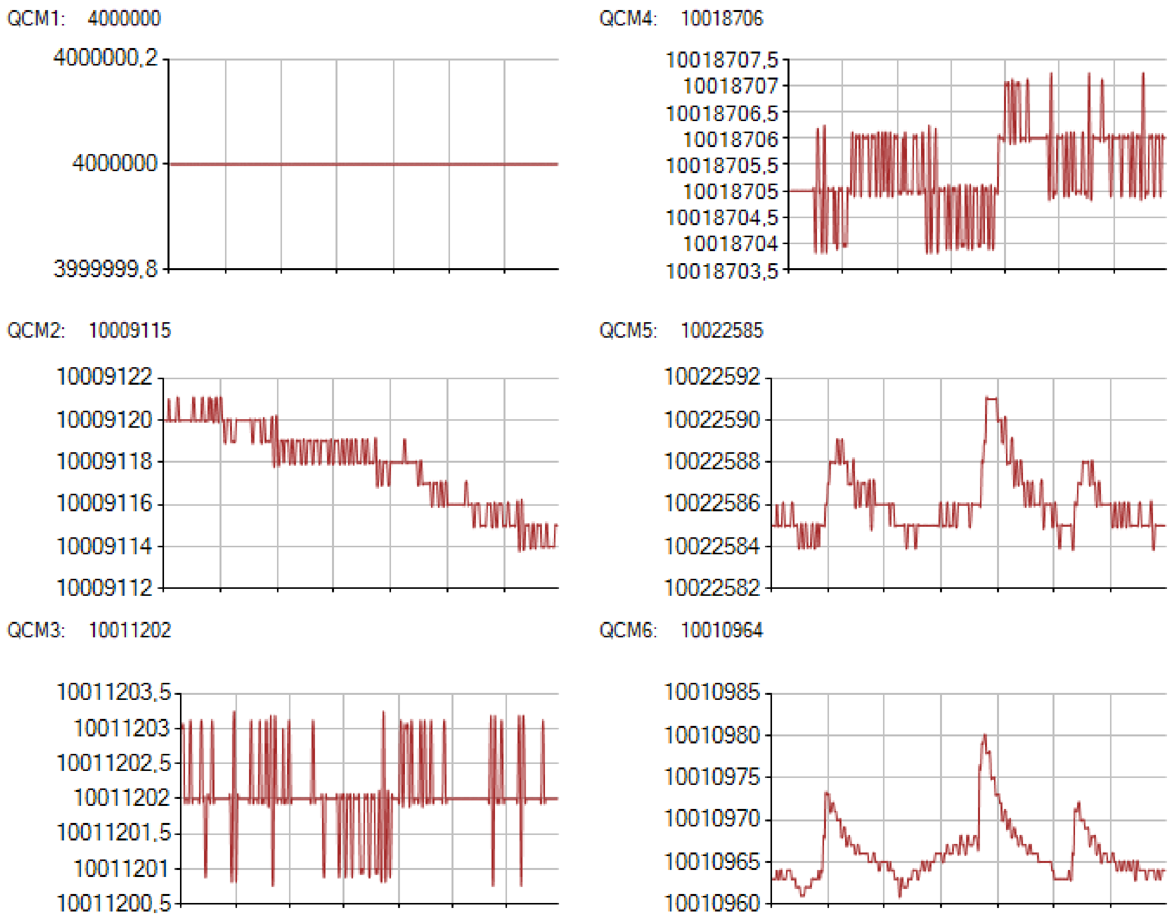

Figure S9. Biosensors' responses to acetaldehyde at the concentration of 856,22 ppm. The graphs were directly taken from the software. QCM 1 → empty slot, QCM 2 → OBPP4 AcO^-, QCM 3 → OBPP4 TFA^-, QCM 4 → OBPP4 GSGSGS TFA^-, QCM 5 → OBPP4 Cl^-, and QCM 6 → OBPP4 GSGSGS Cl^-.

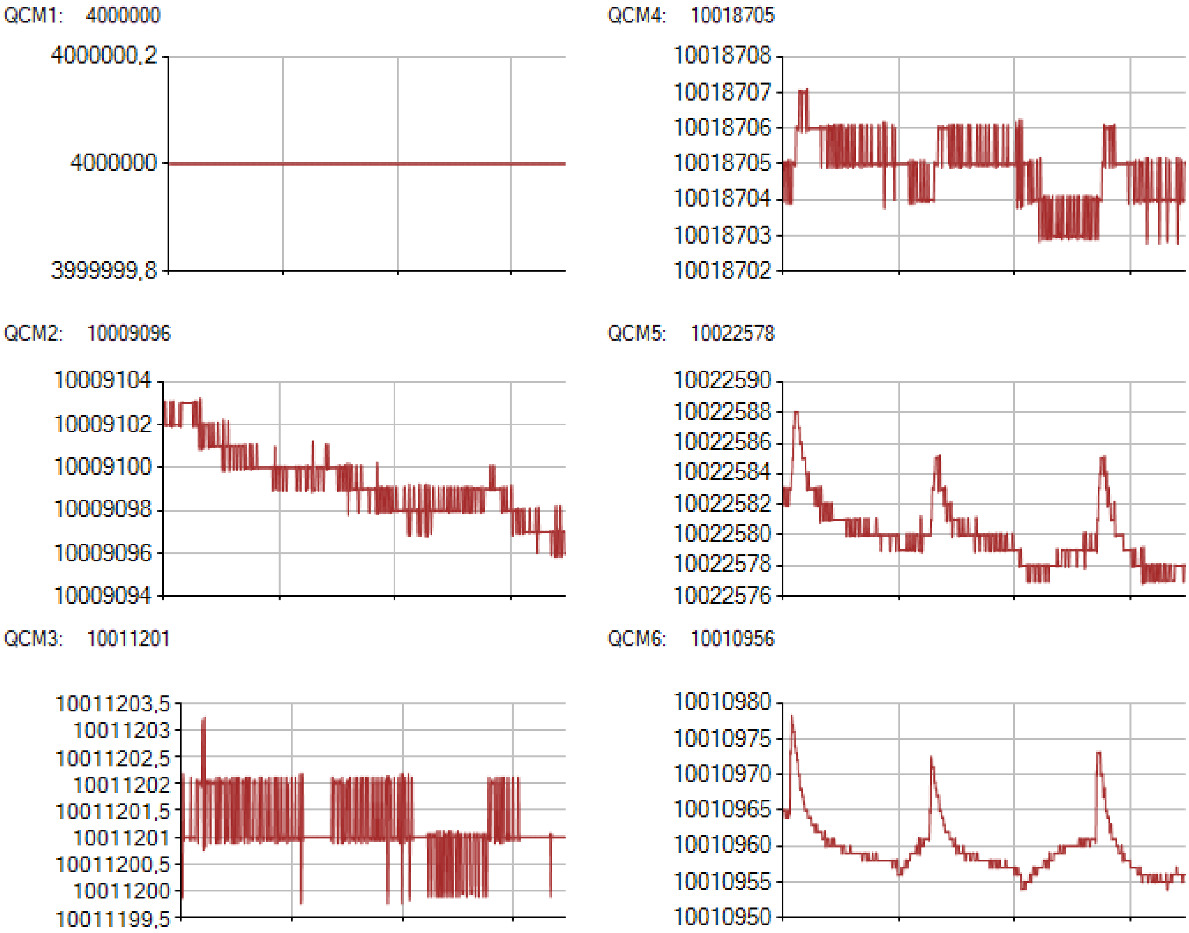

Figure S10. Biosensors' responses to ammonia at the concentration of 277.55 ppm. The graphs were directly taken from the software. QCM 1 → empty slot, QCM 2 → OBPP4 AcO^-, QCM 3 → OBPP4 TFA^-, QCM 4 → OBPP4 GSGSGS TFA^-, QCM 5 → OBPP4 Cl^-, and QCM 6 → OBPP4 GSGSGS Cl^-.

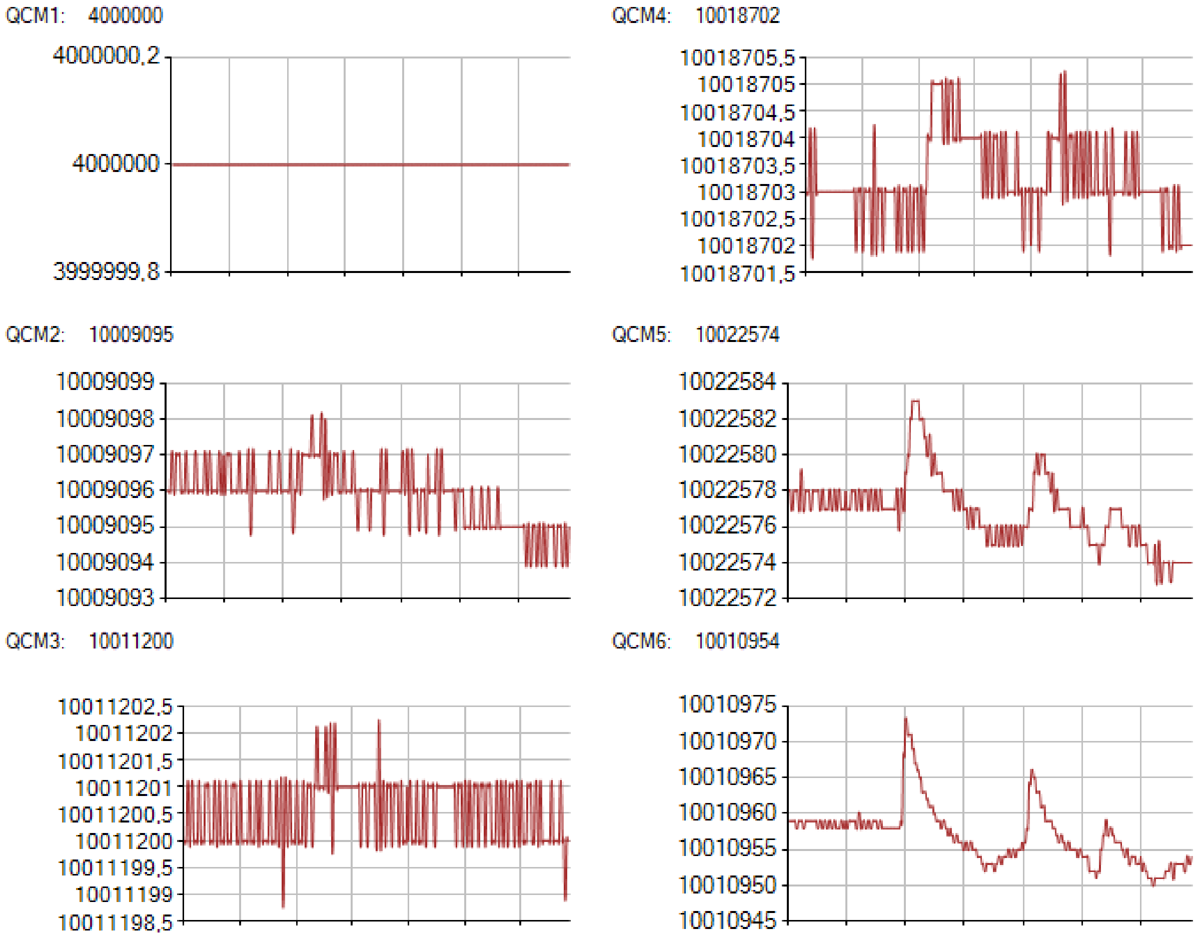

Figure S11. Biosensors' responses to ethyl benzene at the concentration of 398.01 ppm. The graphs were directly taken from the software. QCM 1 → empty slot, QCM 2 → OBPP4 AcO$^-$, QCM 3 → OBPP4 TFA$^-$, QCM 4 → OBPP4 GSGSGS TFA$^-$, QCM 5 → OBPP4 Cl$^-$, and QCM 6 → OBPP4 GSGSGS Cl$^-$

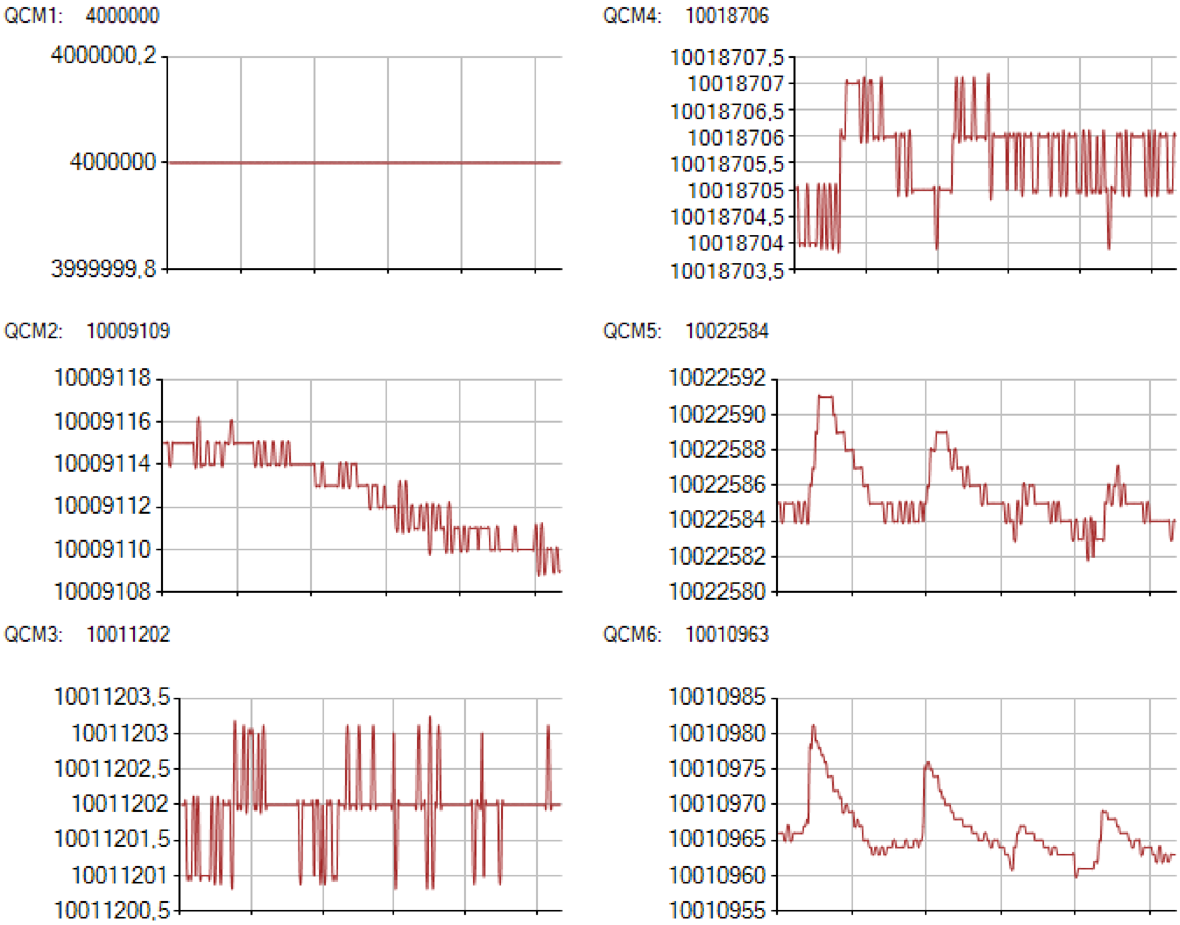

Figure S12. Biosensors' responses to DMS at 640.05 ppm. The graphs were directly taken from the software, QCM 1 → empty slot, QCM 2 → OBPP4 AcO^-, QCM 3 → OBPP4 TFA^-, QCM 4 → OBPP4 GSGSGS TFA^-, QCM 5 → OBPP4 Cl^-, and QCM 6 → OBPP4 GSGSGS Cl^-.

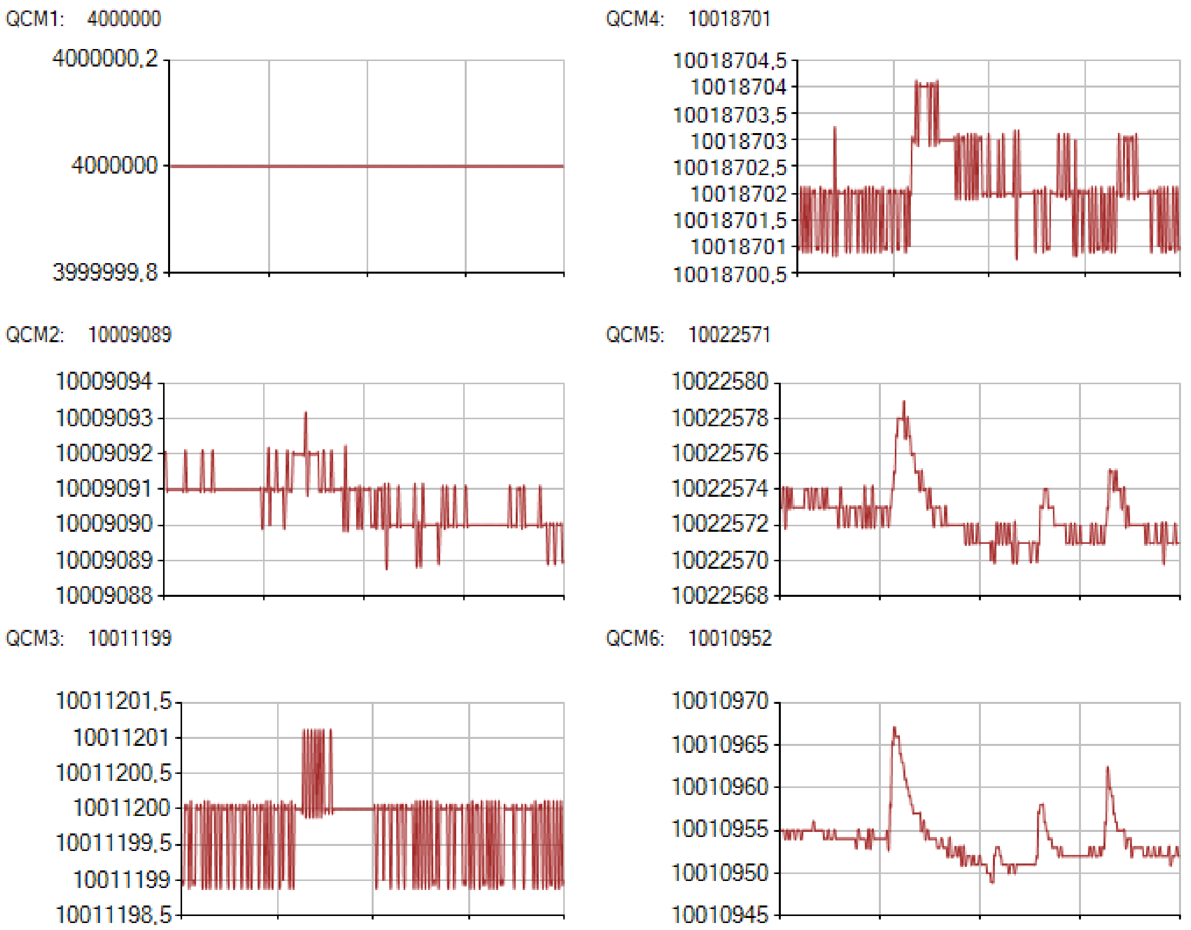

Figure S13. Biosensors' responses to xylene at 389.72 ppm. The graphs were directly taken from the software. QCM 1 → empty slot, QCM 2 → OBPP4 AcO^-, QCM 3 → OBPP4 TFA^-, QCM 4 → OBPP4 GSGSGS TFA^-, QCM 5 → OBPP4 Cl^-, and QCM 6 → OBPP4 GSGSGS Cl^-.

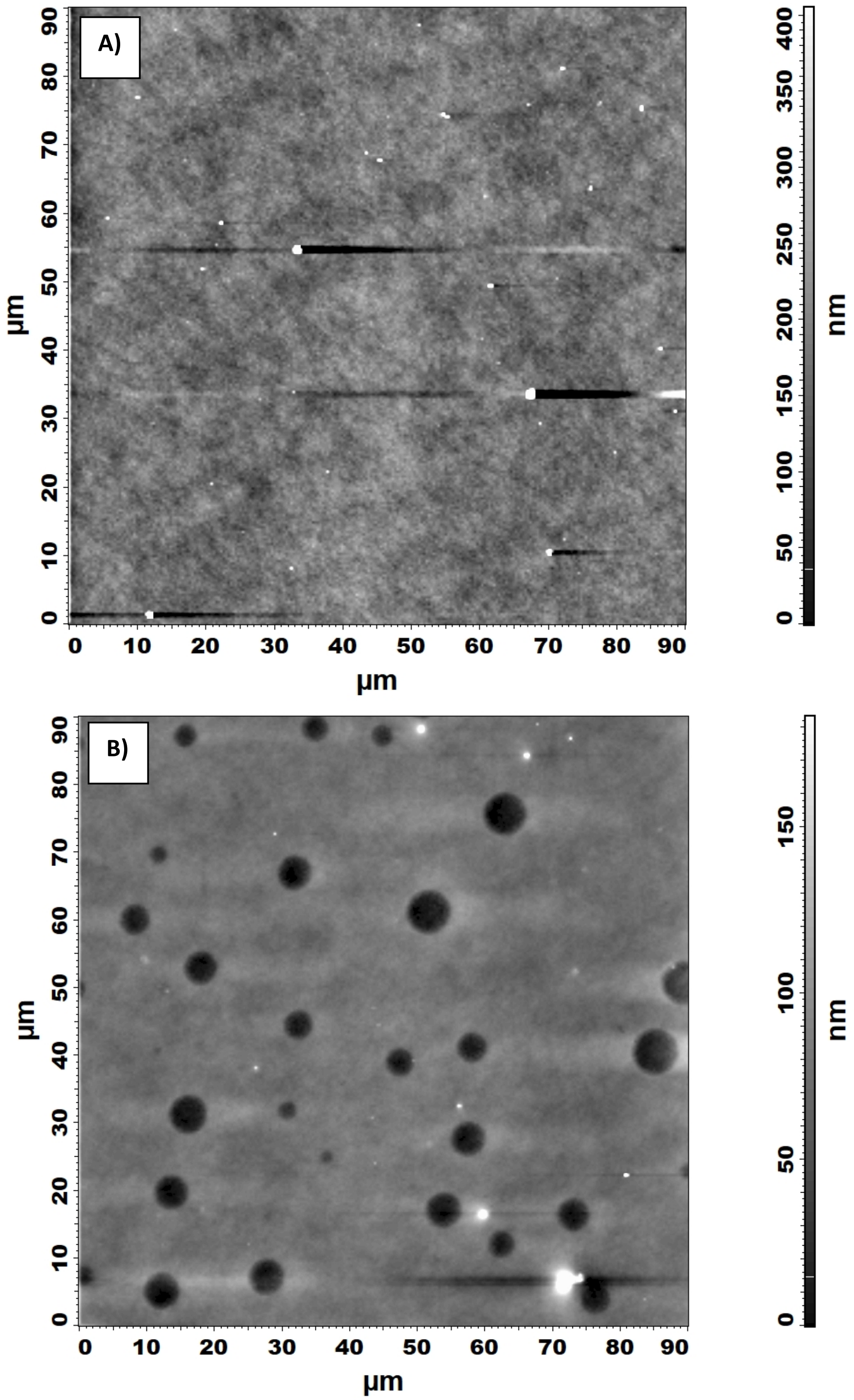

Figure S14. AFM images of biosensors. A) bare QCM sensors (peak-to-peak, Sy: 404.52 nm; ten-point height, Sz: 161.15 nm; and Average Roughness, Sa: 0.93 nm. B) Peptide-based biosensor with linker – GSGSGS with TFA^- counterion (Sy: 183.01 nm, Sz: 72.07 nm, and Sa: 1.34 nm).

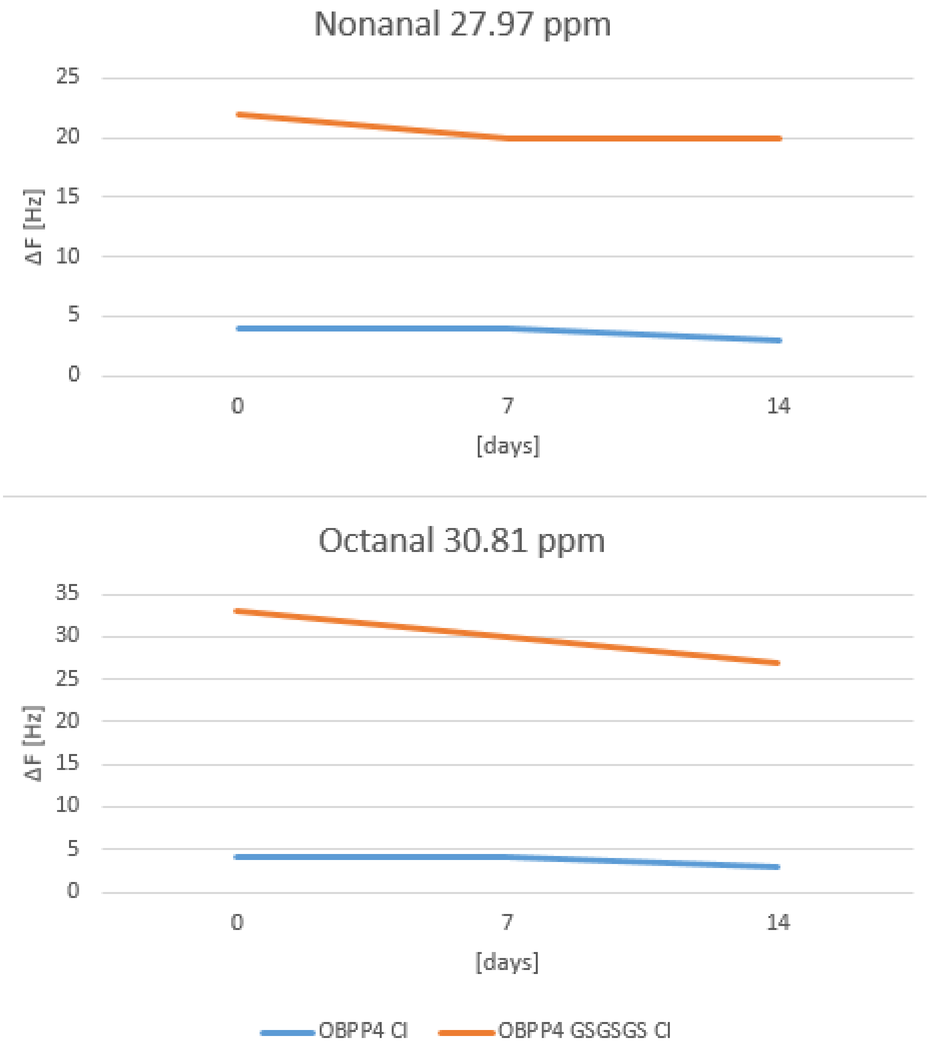

Figure S15. Long-term stability of biosensors under repeated aldehyde exposures. Frequency responses of OBPP4 Cl (blue) and OBPP4 GSGSGS Cl (orange) sensors to nonanal (27.97 ppm) and octanal (30.81 ppm) over 14 days. Data points represent steady-state plateau values from triplicate measurements at each time point.
